# Cost-effectiveness of digital health interventions for supporting mental health of children and young people: a rapid review and narrative synthesis

**DOI:** 10.1007/s00787-025-02932-1

**Published:** 2026-03-04

**Authors:** Amarech Obse, Gabriela Pavarini, Mina Fazel, Minhua Ma, Daisy Fancourt, Craig Morgan, Eli Harriss, Kamaldeep Bhui, Lindsay Smith, Siobhan Hugh-Jones, Sania Shakoor, Georgina Hosang, Laura Havers, Anna Mankee-Williams, Paul McCrone

**Affiliations:** 1 University of Greenwich, London, UK; 2 University of Oxford, Oxford, UK; 3 University of Surrey, Guildford, UK; 4 University College London, London, UK; 5 King’s College London, London, UK; 6 Oxford Health NHS Foundation Trust, Oxford, UK; 7 University of Leeds, Leeds, UK; 8 Queen Mary University of London, London, UK; 9 Falmouth University, Falmouth, UK

**Keywords:** Adverse childhood experiences, Cost-effectiveness, Digital health interventions, Economic evaluation, Mental health, Rapid review

## Abstract

Globally in 2021, about 1 in 4 children and young people (CYP) below age 25 lived with poor mental health. Various forms of digital health interventions (DHIs) have been introduced in attempts to address unmet mental healthcare needs among young people. DHIs use websites or applications in computers, tablets, or smartphones to deliver mental health interventions, which are either self-directed, or therapist guided. This rapid review synthesises the evidence on the cost-effectiveness of DHIs for supporting mental health in CYP. A literature search was carried out in MEDLINE and PsycINFO using Ovid platform. The search was restricted to peer-reviewed studies published in English between Jan 2018 to May 2025. Eligible studies were identified using the population, intervention, comparator, outcome, and study design (PICOS) framework. Studies were eligible for inclusion if they provided a trial- or model-based full economic evaluation or return on investment of a DHI targeting mental health in CYP up to 25 years of age. Titles and abstracts of 1,265 records were screened. 15 studies met the criteria for inclusion following full-text screening of 28 studies. The focal outcomes in the studies included reduction in symptoms linked to depression, anxiety, and alcohol use. Most studies evaluated computerised cognitive behaviour therapy (CBT) or interventions that included aspects of CBT as the primary intervention. All studies except two reported the DHIs to be cost-effective, at least under certain conditions. Two model-based and three trial-based studies reported that the interventions were dominant (cost saving and more effective) with respect to at least one outcome measure. There is some evidence to suggest DHIs hold the potential to expand and extend mental health support for CYP with minimal therapist involvement. Yet, the evidence is not conclusive due to short follow-up periods, variability in the methodological approaches and reporting of results. High quality evidence on cost-effectiveness of DHIs with comparable methodological approaches is needed to inform implementation decisions.

**Supplementary Information:**

The online version contains supplementary material available at 10.1007/s00787-025-02932-1.

## Introduction

In 2021, 26.61% of children and young people (CYP) below the age of 25 years lived with poor mental health, and neurodevelopmental conditions worldwide [1]. These mental health problems include anxiety, depression, eating disorders, and conduct problems, while attention-deficit/hyperactivity disorder (ADHD) and autism spectrum disorder (ASD) are among the most common neurodevelopmental challenges [2]. CYP with neurodevelopmental conditions are more likely to develop mental health problems compared to their neurotypical peers [2, 3].

Poor mental health in CYP can be long-lasting with wide-ranging effects on physical health [4, 5], educational attainment [6, 7], and overall life opportunities [8, 9]. These lifelong effects occur even if mental health problems do not persist [8, 9]. An earlier retrospective study showed that most adult mental health problems are established by age 14 (50%) and age 21 (75%) [10], which is supported by a recent systematic review of prospective studies that showed increased mental health problems in adults who experienced mental health difficulties during childhood and adolescence [11]. CYP with mental health problems are less likely to complete high school, attend college, and graduate from college; the effect being more pronounced in those facing mental health problems at younger ages [8, 9]. As adults they have reduced labour supply, income and probability of being and staying married [8, 9].

Despite the burden of poor mental health, CYPs’ access to mental health treatment is limited [12–14]. A recent systematic review synthesised evidence from 53 studies and reported four main barriers to mental health care for youth: limited knowledge of mental health problems and services (e.g., negative attitude or perception of help-seeking), social factors (e.g., public stigma), perceived quality of relationship with therapists (e.g., ability to trust and confidentiality), and systemic and structural barriers (e.g., cost, logistics, and accessibility of professional help) [14]. Both demand and supply side barriers contribute to the mental health treatment gap. On the supply side, there is a critical shortage of therapists due to low investment in mental health provision, difficulties in training, employment, retention and performance of mental health professionals [15]. A shortage of 11 million mental health workers is projected by 2030 mostly in low- and lower-middle income countries [15]. On the other hand, demand for mental health services has been rising [16] with the pandemic exacerbating the already stretched workforce affecting staff workload, wellbeing and performance [16, 17]. Furthermore, there is substantial disparity in the distribution of mental health resources both between and within countries [18, 19]. For example, in 2020, the global median number of mental health workers per 100,000 population for high income countries was 64 but only 1.4 in low-income countries [18]. The mental health worker density had reduced in 2022 in both high-income (60 per 100,000) and low-income (less than one per 100,000) countries with greater shortage of specialised mental health professionals for children and adolescents (0.01 per 100,000 population) in low-income countries [15].

With advances in technology, digital health interventions (DHIs) have emerged in healthcare systems with potential for reducing the gap between need and provision of care, generating clinical benefits [20]. DHIs use websites or applications in computers, tablets, or smartphones to deliver mental health interventions, which are either self-directed or therapist-guided [21]. DHIs can be designed for providing mental health prevention and promotion services [22] or treatment of existing mental health problems [20]. Preventive DHIs include various activities aimed at reducing the occurrence of mental health problems and/or timely help seeking such as psychoeducation, mindfulness practices, problem solving, coping skills training and help seeking behaviour [23–25]. With regards to DHIs for treatment, the form of digital intervention that has been most widely researched is internet-delivered cognitive behavioural therapy (ICBT) [21, 26, 27], typically delivered via a web portal or an app. However, other modalities have also been investigated including games, virtual or augmented reality [27–31], and other approaches such as mindfulness, problem-solving, and dialectical behaviour therapy [32].

There is a growing body of literature on effectiveness and cost-effectiveness of DHIs for the prevention and treatment of mental health problems in CYP, but the evidence is mixed [20, 33]. In the case of prevention, a meta-analysis showed medium-to-low effectiveness of the DHIs in promoting mental health in adolescents [33]. Similarly, a meta-review of effectiveness of DHIs for mental health (prevention and treatment) in children and adolescents suggested effectiveness of DHIs although definitive conclusions could not be made owing to methodological differences [20]. Another systematic review of the effectiveness of DHIs in CYP aged 10–24 corroborated the evidence from the previous reviews, however, none of the studies included in the review reported cost-effectiveness of the DHIs [34]. Recently, some evidence on the cost-effectiveness of DHIs has emerged which was synthesised in a meta-analysis and in a separate systematic review [35, 36]. These reviews included evidence for combined CYP and adult populations and reported that DHIs were likely to be cost-effective but heterogeneity and high risk of bias in the included studies limited conclusive evidence [35, 36]. However, these reviews did not provide evidence of cost-effectiveness separately for CYP and adult population. Among the methodological issues limiting conclusive evidence from randomised trials are small sample sizes, variation in the content and mode of delivery of DHIs, short-term follow-up, and low levels of engagement and uptake [20, 28, 37]. Furthermore, despite their scalable nature, digital interventions can be costly to develop, maintain, and deploy, especially those requiring high-end gaming graphics, virtual reality (VR), or augmented reality (AR) equipment. Finally, while some DHI are designed for standalone use, others require involvement and training from a clinician, which can incur considerable cost [26]. As the field grows, it is crucial to carefully assess the cost-effectiveness of this approach in different population groups.

While DHIs have the potential for addressing unmet healthcare needs, “the opportunity cost of investment is also large and often sunk” [38, p. 4.] In settings where DHIs are paid out of healthcare budgets, cost-effectiveness studies of the DHI can inform whether investment in DHIs provide good value for money compared with alternative courses of action [38, 39]. To our knowledge there are no systematic reviews of cost-effectiveness of DHIs focusing on mental health of CYP. To this end, the purpose of this rapid review is to synthesise evidence on cost-effectiveness of DHIs for treatment, promotion, and prevention of mental health problems (also neurodevelopmental and substance use problems) of CYP (up to age 25 years). The existing reviews [35, 36] presented evidence of cost-effectiveness for combined CYP and adult population. It is necessary to conduct a separate synthesis of the cost-effectiveness of DHIs in CYP since costs and effects of DHIs can vary between CYP and adult population. Furthermore, the existing reviews have included all types of DHIs including treatment provided remotely by therapists (e.g. telepsychiatry). In this review, DHIs with minimal therapist involvement will be included. A rapid review was conducted (in place of a systematic review) due to time and other resource limitations. The review includes both trial- and model-based economic evaluations. While trial- and model-based economic evaluation are not comparable, model-based economic evaluations are included to appraise long-term effectiveness of DHIs since trial-based intervention are usually limited to a short-term assessment of cost-effectiveness. Neurodevelopmental and substance use problems are included in this review because many children and adolescents with these conditions have mental health comorbidities and receive care at mental health clinics [2, 3, 40, 41].

## Method

### Search strategy

The review follows Preferred Reporting Items for Systematic Reviews and Meta-Analysis (PRISMA) reporting guidelines [42].

Ovid MEDLINE and Ovid PsycINFO were searched on 24/10/2023 (and updated in full on 06/05/2025) for English language studies published from 2018 to the search date (to capture the most recent evidence). The searches were conducted by an information scientist (EH). Ovid MEDLINE was searched using adapted versions of previously published search strategies for the four concepts: the CADTH Economic Evaluations & Models MEDLINE^1^ filter was used to retrieve economic evaluations; records about health apps (DHIs) were retrieved using the Ayiku et al. [43] filter; and records about children and young people, and mental health, were retrieved using terms from the systematic review by Bear et al. [44]. The search strategy was then translated accordingly for Ovid PsycINFO. The full strategies and search terms are available in Supplementary Material 1. All references were exported to EndNote 20 (Thomson Reuters, New York, NY), deduplicated manually, and were exported to Rayyan (www.rayyan.ai) for screening.

### Selection criteria

The eligibility criteria are presented in Table 1 based on the population, intervention, comparator, outcome, and study design (PICOS) framework [45]. Studies were eligible for inclusion if they included an economic evaluation of a DHI designed to support the mental health of CYP aged up to 25 years. Eligible interventions were DHIs developed to provide therapies, treatments, and/or tools to improve poor mental health and/or quality of life delivered using computer or smartphone platforms. The interventions could be delivered as text, narration, talking therapies, or video games to which users were given access to read, view or download materials. The DHI could involve contact with a therapist synchronously or asynchronously where therapists provided guidance, responded to general questions and/or provided feedback. Parents could be involved in the intervention or assist their child(ren) using the digital technology. Treatments fully provided by therapists remotely using digital technologies (such as telepsychiatry) were excluded. Studies with participant level and model-level data were both eligible for inclusion. Trial-based economic evaluations were included if there was at least one comparator (e.g., usual care or another active intervention). Pre-post studies were excluded unless there was a comparator arm (e.g. multi-arm pre-post study) since it is not possible to attribute outcomes of treatment to an intervention without sufficient control. Return on investment and social return on investment studies were also eligible for inclusion if there was a comparator. Economic evaluation studies may include cost-effectiveness analysis (CEA), cost-utility analysis (CUA), cost-benefit analysis (CBA), cost-consequences analysis, and cost-minimisation analysis (if outcomes are shown to be equal). The difference in these economic evaluation methods arises from how treatment outcomes are measured. Outcome is measured using natural or clinical units in CEA (e.g., Social Anxiety Scale (SAS) for anxiety), quality-adjusted life-years (QALYs) in CUA, and monetary values in CBA [46]. Reviews, reports, editorial, commentary, conference papers, protocols, letters, and books were excluded.

**Table 1:** PICO eligibility criteria

Population (*P*)	Children and young people (CYP) (up to 25 years of age) with or without mental health problems from the general population or CYP with mental health problems selected from clinical setting. Mental health problems in CYP includes clinical or self-reported poor mental health including depression, generalised anxiety, eating disorders, panic disorder, conduct problems, or any other type of distress and/or CYP with neurodevelopmental conditions such as attention-deficit/hyperactivity disorder (ADHD), autism spectrum disorder (ASD), obsessive compulsive disorder (OCD), learning difficulties, etc. Studies in CYP and adults if outcomes for CYP are reported separately.
Intervention (I)	Mobile application or web-based digital health technologies used to deliver mental health intervention through text messages, illustrations, videos, narration, games, among others.
Comparator (C)	No intervention, treatment as usual, or any other active digital or non-digital intervention.
Outcomes (O)	Mental health and/or wellbeing measured using validated scales for CYP such as Social Anxiety Scale (SAS), Patient Health Questionnaire (PHQ), quality-adjusted life-years (QALYs), health-related quality of life (HRQoL) and/or appropriate measures of outcomes for neurodevelopmental conditions.
Study design (S)	Randomised controlled trials (RCTs) or cluster randomised trials (cRCT) or controlled trials or quasi-experimental design or model based economic evaluations. The types of economic evaluations included are CEA, CUA, CBA, return on investment, and social return on investment.

### Data extraction and analysis

The publications retrieved from database searches were imported into Rayyan software for screening. One reviewer (AO) screened the title and abstracts of all records while the second reviewer (PM) independently screened 10% of the records to confirm that the eligibility criteria had been consistently applied. AO screened all full-text publications, and the second reviewer (PM) checked the included and excluded full-text articles. A spreadsheet was developed for data extraction which included items on study characteristics (author, year, country, design), population, intervention(s), comparator, evaluation type, perspective, time horizon, year of pricing, discount rates, costs, outcomes, and main conclusions. AO extracted data from the included studies and the PM checked the accuracy of extracted data. Any differences were resolved through discussion. The summary of characteristics of the studies, interventions, treatment outcomes and cost-effectiveness results are presented in Table 2.

**Table 2:** Characteristics of studies included in the review

Author(s), year, country, citations	Study type	Mental health/neuro-developmental conditions	Study population, recruitment	Intervention: contents, number of sessions, delivery methods and location	Comparator: contents, number of sessions, delivery methods and location	Time horizon, perspective, discount rate	Effects, incremental effectiveness/QALY (IE)	Costs/, incremental cost (IC) in US$ 2024	ICER/ICUR in US$ 2024, WTP threshold (US$), cost-effectiveness	Quality
Andrén et al. [47], Sweden	Trial based, CEA, CUA	Tourette’s syndrome (TS) and chronic tic disorder (CTD); primary measure of effectiveness: Total Tic Severity Score of the Yale Global Tic Severity Scale (YGTSS-TTSS)	9–17 years; 221 participants; 111 in intervention, 110 in comparator recruited from clinical referral and self-referral	Internet-delivered exposure and response prevention (ERP) emphasising response prevention and exposure therapy presented through texts, illustrations, videos, worksheets, exercises, and homework assignments; parents were provided with separate access containing overlapping treatment content. The intervention was delivered online over 10 weeks.	A therapist-assisted online educational intervention developed to mirror the ERP program, excluding its central exposure element. The content covered TS, CTD, comorbid conditions, and behavioural exercises, delivered online over a 10-week period.	3 months; health facility, health sector, societal perspective	Significant reductions in tic severity (YGTSS-TTSS) in both groups: ITT IE (95% CI) = −0.53 (−1.28; 0.22); IE in QALY = 0.003 (−0.004; 0.010)	IC (95% CI): health facility = 17.18, health sector = 103.58 (−73.15; 513.16), society = 30.13 (−458.87; 1,108)	ICER per treatment response: 89 (health facility), and 540 (health sector), 156.57 (societal); ICUR per QALY: 6,235 (health facility) and 37,596 (health sector), 10,938 (societal); WTP 79,000; 66%−76% probability of ERP being cost-effective depending on perspective	72%
Andrén et al. [48]; Sweden	Trial based, CEA, CUA	Tourette’s syndrome (TS) and chronic tic disorder (CTD); CEA outcome measure: Total Tic Severity Score of the Yale Global Tic Severity Scale (YGTSS-TTSS)	9–17 years; 221 participants, 111 in intervention, 110 in comparator recruited from clinical referral and self-referral	Internet-delivered exposure and response prevention (ERP) emphasising response prevention and exposure therapy presented through texts, illustrations, videos, worksheets, exercises, and homework assignments; parents were provided with separate access containing overlapping treatment content. The intervention was delivered online over 10 weeks.	A therapist-assisted online educational intervention developed to mirror the ERP program, excluding its central exposure element. The content covered TS, CTD, comorbid conditions, and behavioural exercises, delivered online over a 10-week period.	12 months; health facility, health sector, societal perspective	Significant reductions in tic severity (YGTSS-TTSS) baseline to 12 months: effect size = 0.49 (0.34; 0.64) (intervention) and 0.50 (0.33; 0.67); (comparator); QALY 1.016 (SE = 0.027) (intervention), 1.000 (SE = 0.013) (comparator)	IC (95% CI): health facility = 17.18 (5.76; 28.59); health sector = −95.85 (−499.42; 1,109); society = 144.83 (−1,204; 2,907)	ICER: 334.69 (health facility), dominant (health sector), and 2,818.17 (societal); ICUR: 2,552.69 (health facility), dominant (health sector), and 20,561.10 (societal); WTP 79,000; ERP dominant or 65%−78% probability of being cost-effective depending on perspective	74%
Aspvall et al. [49], Sweden	Trial based, CEA, CUA	Obsessive-compulsive disorder (OCD) as primary diagnosis; CEA outcome measure: treatment response	8–17 years; 152 participants: 74 in G-ICBT, 78 in in-person CBT recruited from specialist paediatric OCD clinics and self-referral	G-ICBT with stepped care: covering psychoeducation, exposure and response prevention, and relapse prevention, with a dedicated module for parents delivered in parallel; in-person CBT for non-responders at the end of 3-month; 14 modules over 16-weeks delivered online	In-person CBT: a therapist delivered manualized CBT; longer sessions and home visits based on need; additional face-to-face therapy for non-responders at end of 3-months; 14 sessions of CBT over 16-weeks; delivered at specialist paediatric OCD clinics	6 months; health professional, health sector, and societal perspective	IE (95% CI) in treatment response = 0.0004 (−0.151; 0.152); incremental QALY = −0.029 (−0.055; 0.006)	ITT: IC (95% CI) = −1,814 (−2,461; −697) health sector, −2,072 (−2,950; −573) societal perspective; PP IC (95% CI) = −2,001 (−2,529; −1,090) for health sector, – 2,043 (−2,823; −713) for society	WTP threshold and ICER not provided. G-ICBT was less costly (with about the same treatment response) than in-person CBT based on treatment response; based on QALYs, G-ICBT was less costly and less effective for all 3 perspectives	78%
Deluca et al. [50], England	Trial based, CEA, CUA	Alcohol consumption; CEA outcome measure: average alcohol consumption in standard UK units (AUDIT-C)	14–17 years low risk adolescents recruited from emergency departments	Two arms: (1) Personalised Feedback and Brief Advice (PFBA) only and (2) PFBA + smartphone-based (eBI). Both provided feedback on screening results and alcohol use advice. The eBI offered additional online support via a gamified app introduced in one 20-minute session, followed by self-directed use.	Screening only group (treatment as usual) at Emergency Departments	6 and 12 months; societal and National Health Service-Personal Social Services (NHS-PSS) perspective	Mean differences in AUDIT-C score between the intervention group and control was − 0.01 (−0.12; 0.11) for both interventions; QALYs were plotted in CEAC curve)	Costs for each intervention or IC were not given only plotted in the CEAC curve	WTP threshold of 28,777 to 43,165; ICERs for PFBA were 250,499 (societal) and 231,377 (NHS-PSS) per QALY gained; from societal perspective effectiveness probability for PFBA varied 26%−33%; eBI had 9% probability of being cost-effective based on either perspective	68%
Jolstedt et al. [51], Sweden	Trial based, CEA, CUA	Principal Anxiety Disorder; CEA outcome measure: Clinician Severity Rating (CSR)	8–12 years; 131 participants, 61 in ICBT and 65 in internet delivered child-directed play (ICDP); recruited nationally	ICBT delivered via text, illustrations, videos, and exercises; includes separate parent program; 12 sequential modules (one per week); online delivery	ICDP: parents engage in non-directive, praise-based play to enhance the parent–child relationship; ~20 min, 3–4 times per week; home-based	3 months; Societal perspective	CSR improved significantly in both groups; ICBT group improved more than ICDP group. Mean difference (95% CI) in CSR = 0.53 (0.12; 0.87); difference in QALYs = 0.02 (0.02; 0.02)	Difference in mean total societal cost (95% CI) = 695.36 (672.96; 717.74)	ICER (95% CI: −2,026 (−2,106; −1,947), 80% probability of ICBT being cost-effective if WTP more for ICBT was zero; 100% probability of ICBT being cost-effective if WTP was 5,370; ICBT was not cost-effective when QALYs were used	78%
Kling et al. [52], Norway	Trial-based CUA	Psychological distress (due to visible facial or bodily appearance/difference)	age 11–18; 102 participants: 55 in intervention, 47 in waiting list control	Young Person’s Face (YPF): web-based psychosocial intervention featuring activities on coping and adaptation strategies; seven weekly 30–40-minute sessions plus a booster; online delivery	Care as usual: usual care with no standardized psychosocial program; support provided as needed through available services, e.g., routine consultations at local hospitals	3 months; provider	Adjusted QALYs (95% CI) = 0.205 (0.200; 0.210) in intervention, 0.201 (0.196; 0.206) in comparator; IE = 0.004	Cost of the control arm was assumed zero due to unavailability of any services for the conditions under study; cost for intervention 37.00 per patient per year. IC = 37.00 assuming 40 users per year	ICUR was 9,418 per QALY; the intervention has 83% probability of being cost-effective as compared to comparator at WTP threshold of 50,400.	67%
Le et al. [53], Australia	Trial-based, CUA	Any mental distress	18–25 years; 413 participants 205 in intervention, 208 in comparator	Internet-based mental health help-seeking intervention (‘Link’): four steps – select symptoms, rate severity, indicate preferred type of help, choose service and view additional information including costs	Usual online and offline strategies to seek help.	3 months; provider	ITT: incremental effect (95% CI) = 0.01 (0.01; 0.02)	ITT: incremental cost (95% CI) = −70.84 (−306.69; 120.17)	WTP was 19,755 per QALY; ICUR (ITT): dominant; ICUR (PP): dominant; 95% probability of being cost-effective if willingness-to-pay is over 7,047 per QALY gains	67%
Lee et al. [54], Australia	Model based, CUA	Anxiety	11–17 year; 30 (45.7%) schools comprising 1,477 (59.0%) students were involved in the effectiveness trial.	Internet-based e-health intervention for anxiety (MoodGYM): CBT-based online program – five modules delivered via text, animations, and interactive exercises; one 20–40 min module per week in class; teacher-supervised	‘No intervention’ scenario: MoodGYM intervention was not delivered across schools	6 months initial effectiveness analysis: effect modelling time horizon 10 years; partial societal perspective	MoodGYM intervention prevented around 10,500 anxiety cases. IE (95% CI) = 842 (289; 1,681) QALYs over 10 years	IC (95% CI) = −11.28 million (−38.20 million; 1.51 million)	WTP threshold 35,261 per QALY; ICUR was found to be dominant when compared to the comparator; 100% probability of being cost-effective; return on investment was 16.7 million; for every Aus$ 1 (US$ 0.89) invested there was Aus$3.06 (US$ 16.7) return on investment	85%
Nordh et al. [55], Sweden	Trial based, CEA, CUA	Social anxiety disorder; CEA outcome measure: Clinician Severity Rating (CSR) score	10–17 years; 103 participants, 51 in ICBT, 52 in ISUPPORT recruited from referrals by healthcare professionals and self-referrals	Therapist-guided ICBT: child and parent online modules (psychoeducation, exposure, social skills, focus-shifting) over 10 weeks; 20–30 min therapist video calls at weeks 3, 5, and 7	ISSUPORT: child and parent supportive online therapy which includes psychoeducation, interpersonal relations, and healthy habits; excludes active CBT components; therapist video sessions (20–40 min) at weeks 3, 5, and 7	3 months; health professional and societal perspective	ICBT moderately effective than ISUPPORT using the Clinician Severity Rating (CSR); IE (95% CI) in CSR = −0.62 (−1.04; −0.20); IE in QALYs = −0.011, t = −0.55, *p* = 0.58	Societal cost of over three months 3,568 (2,655; 4,480) in intervention; and 5,151 (3,740; 6,562) in comparator	ICUR not provided “due to minimal difference in QALYs”. ICER (95% CI) was − 2,6324 (- 28,667; −23,981) from societal and 827 (750; 904) from health professional perspectives. WTP threshold wasnot provided.	72%
Vargas-Martínez et al. [56], Spain	Trial based, CEA, CUA	Binge drinking (BD): CEA outcome measure: reduction in the number of BD occasions over 30 days	15–19 year; 367 participants from 15 public high schools; 210 in intervention, 157 in comparator	Alerta Alcohol Program: web-based, computer-tailored intervention: 6 sessions with personalized information, preventive messages, feedback, and benefits of not drinking; baseline questionnaire at session 1, repeated at session 6 (4 months later)	Waiting-list control: baseline and follow-up questionnaires at 4 months only; no active intervention	4 months; NHS and societal perspective	IE, BD occasions = 0.035; incremental QALY = 0.008	IC (NHS) = 1.102; IC (societal) = −529.13; (SD not provided)	WTP threshold: 32,012 to 36,585. ICER and ICUR per BD occasions were 31.64 and 135.18 respectively (NHS perspective); ICER and ICUR dominant from societal perspective.	78%
Wasil et al. [57], Kenya	Trial-based, CEA	Depressive symptom; CEA outcome measure: Patient Health Questionnaire (PHQ)	13–18 years; 103 students from a single private school; 50 in intervention; 51 in comparator	Shamiri-Digital: online single-session self-help (growth mindset, gratitude, value affirmation); 60 min, completed in one sitting	Active online comparator on study skills: two modules on note-taking and study habits; 60 min, single session; matched in structure to the intervention	2 weeks; school, researcher, and societal perspective	PHQ-9 depression scores, 39% (intervention) and 14% (comparator) experienced clinically meaningful improvement over 2 weeks’ time	Cost assumed to be the same for the intervention and comparator. Cost per student 4.23 (societal), 1.70 (school), 2.54 researcher perspective	The average effectiveness-cost ratio was 0.5 (SD = 1.47) (intervention) and − 0.06 (SD = 1.26) (comparator); i.e., 0.5-point reduction per dollar spent (intervention); no improvement (comparator)	65%
Wright et al. [58], England	Trial-based CUA	Low mood/depression	12- to 18-year-old; 70 in intervention, 69 in comparator	Computerised cognitive–behavioural therapy (CCBT) (Stressbusters): eight-session CCBT program (30–45 min each) with interactive videos, animations, graphics, printouts, and homework; sessions completed in sequence	Self-help websites on wellbeing and mood (text, narratives, videos); participants selected order/content; no homework	12 months; provider perspective	QALY (95% CI) over 12 months: 0.622 (0.514; 0.729) in intervention; 0.623 (0.521; 0.725) in comparator; IE (95% CI) = 0.03 (−0.09, 0.14)	IC (95% CI) = −27.77 (−458.46; 402.92)	ICER negative due to lower costs and higher QALYs (not reported), the probability that the CCBT was cost-effective was estimated at about 65% if the WTP is between 28,694 and 43,042 per QALY.	63%
Creswell et al. [59]; UK	Trial based; CUA, CEA	Anxiety; CEA outcome measure: Child Anxiety Impact Scale–parent report (CAIS-P)	5–12 years; participants recruited during their usual clinic visit; 222 in intervention, 221 in comparator	OSI plus therapist support (OSI + TS): parent-led CBT with therapist support; 7 modules (text, audio, video) with exercises; optional child game app; 20-min biweekly therapist sessions; review 4 weeks post-intervention	Treatment as usual	14 weeks and 24 weeks; NHS and Personal Social Services (PSS)	QALYs (SE) over 26 weeks: based on UK adult value sets = 0.428 (0.004) for OIS + TS and 0.443 (0.004) at TAU; IE (95% CI) = −0.007 (−0.015; 0.002). Based on Australian (AU) adolescent value sets: 0.331 (0.008), 0.347 (0.008), IE = −0.002 (−0.017; 0.012), respectively	ITT IC (95% CI) in overall NHS & PSS = −142.86 (−412.68; 126.95)	WTP of 25,615 to 38,422; ITT ICUR was 21,433 (UK adult value set), 63,045 (AU adolescent value set); probability of cost-effectiveness: 30% & 24% (UK adult value set), 60% & 53% AU adolescent value set	78%
Morrish et al. [60]; UK	Trial-based; CUA	Self-harm	12–17 years old, receiving treatment from specialist for self-harming at least twice in previous one year; 85 in each arm	Usual Care plus BlueIce (UC + BI): a self-help smartphone app with personalised mood-lifting strategies (CBT & DBT-based), 24/7 access, with crisis emergency contact routing	Usual Care (UC): standard care via face-to-face or remote care from specialist clinicians at child and adolescent mental health services (CAMHS)	12-weeks and 6-months; NHS and PSS perspective	QALYs (SE) at 12-weeks and 6 months = 0.156 (0.002) and 0.337 (0.004) for UC + BI; 0.151 (0.002) and 0.335 (0.004) for UC. IE (6 months) = 0.003 (−0.005; 0.017) QALYs; IE (12 weeks) = 0.004 (−0.001; 0.010) QALYs	IC (12 weeks) = −394.31 (- 1,455.0; 666.40) IC (6 months) = −1,139.45 (- 2,807; 528.07)	WTP threshold 28,777 to 43,165; using 28,777 threshold net monetary benefit was estimated at 520.55 (12-weeks) and 1,234 (6-months); the probability of cost-effectiveness was 75% (12-weeks) and 70% (6-months)	83%
Natsky et al. [61]; Colombia	Model-based; CUA	Mental illness	Young people: four age groups included in the model: 0–6, 7–11, 12–17, 18+	9 mental health interventions: school-based (2), community-based (3), training (2), and online interventions (2) modelled individually and in combination; the online interventions were suicide helplines and online (self-help) mental health services	Business as usual (BAU): existing programs and policies remain in place	10 years; healthcare perspective; 5% discount	IE (95%CI) of online mental health services: 10,164 (8,549; 11,870) QALYs; suicide helplines: 579 (31; 1,070) QALYs	IC of online mental health services = −1,930,913 (- 2,427,961; −1,499,287); suicide helplines = 3,433,516 (2,729,608; 4,153,919)	CE threshold: 4,890 per QALY); online mental health service was dominant with INMB 55.13 (45.59; 63.62); ICUR for suicide helplines 5,932	85%

Cost-effectiveness was evaluated by comparing an incremental cost-effectiveness ratio (ICER) or an incremental cost utility ratio (ICUR) with a cost-effectiveness threshold/willingness to pay (WTP) threshold [46]. ICER/ICUR is a ratio of difference in average costs (incremental cost, IC) and difference in average outcomes (incremental effect, IE) between an intervention and a comparator [46]. A WTP threshold represents the maximum amount a country considers to represent value for money and thus willing to pay for an intervention per unit of effect [62]. A DHI was judged as cost-effective, in this review, if the DHI, compared to a comparator(s), was (i) dominant (more effective and less costly), or (ii) the ICER/ICUR per QALY was below the WTP threshold used in the study, or (iii) the probability of cost-effectiveness was greater than 60%.

Only narrative and descriptive syntheses are provided based on perspectives adopted due to methodological differences across the studies. A perspective in economic evaluation refers to the viewpoint adopted for analyses of costs and benefits of an intervention, such as, patient, household, provider, payer, health sector, or societal perspective [63]. Perspectives are crucial for interpreting economic evaluation results since the costs and outcomes analysed depend on the adopted perspectives. For instance, a patient perspective includes costs and benefits from patient’s viewpoint only while a provider perspective includes all costs paid by the health care provider but not any costs borne by patients or society at large. Societal perspective is the broadest with all costs and benefits included in appraisal regardless of who pays for it or benefits from the intervention (patients, providers, purchasers). Societal perspective can also potentially include productivity losses related to the use of health services and costs incurred by other sectors relevant to the intervention [63]. Usually, multiple perspectives (more than one) are adopted in studies.

For ease of interpretation, all costs and ICER/ICUR were adjusted for inflation and converted to 2024 US$ using purchasing power parity (PPP) exchange rates [64]. WTP thresholds are converted to US$ (if reported in another currency) using market exchange rates for the study period rather than PPP exchange rates since WTP thresholds usually remain constant for many years across countries [65, 66].

Given methodological differences, the evidence from trial- and model-based economic evaluations was synthesised separately. Trial-based economic evaluation analyses may follow intention to treat (ITT) and/or per protocol (PP) approaches. The ITT approach investigates cost-effectiveness of an intervention regardless of whether the study sample adheres and completes the trial; in contrast, PP analysis investigates the cost-effectiveness in the sample that have received and completed the treatment without deviations from the protocol [67].

### Risk of bias assessment

We used the Consensus Health Economic Criteria (CHEC) checklist to appraise the reporting quality of the economic evaluations of the included studies [68]. For a rapid review, the risk of bias assessment can be limited to the most important outcomes [69]. Thus, we used CHEC 2013 with 20 items instead of CHEC 2022 with 28 items [70]. The checklist includes items such as reporting target population, objective, time horizon, and appropriateness of measurement and valuation of cost and outcome. Each of quality assessment questions were rated as ‘yes’, ‘no’, ‘unclear’ or ‘not applicable’. The overall reporting quality score for each study was calculated as the ratio of number of ‘yes’ score to the sum of ‘yes’, ‘no’, and ‘unclear’ scores. Based on the total scores, quality was rated as ‘low’ (< 60%), ‘moderate’, (60%−80%), or ‘high’ (>80%) (Supplementary Material 2).

## Results

### Study characteristics

**Fig. 1:**
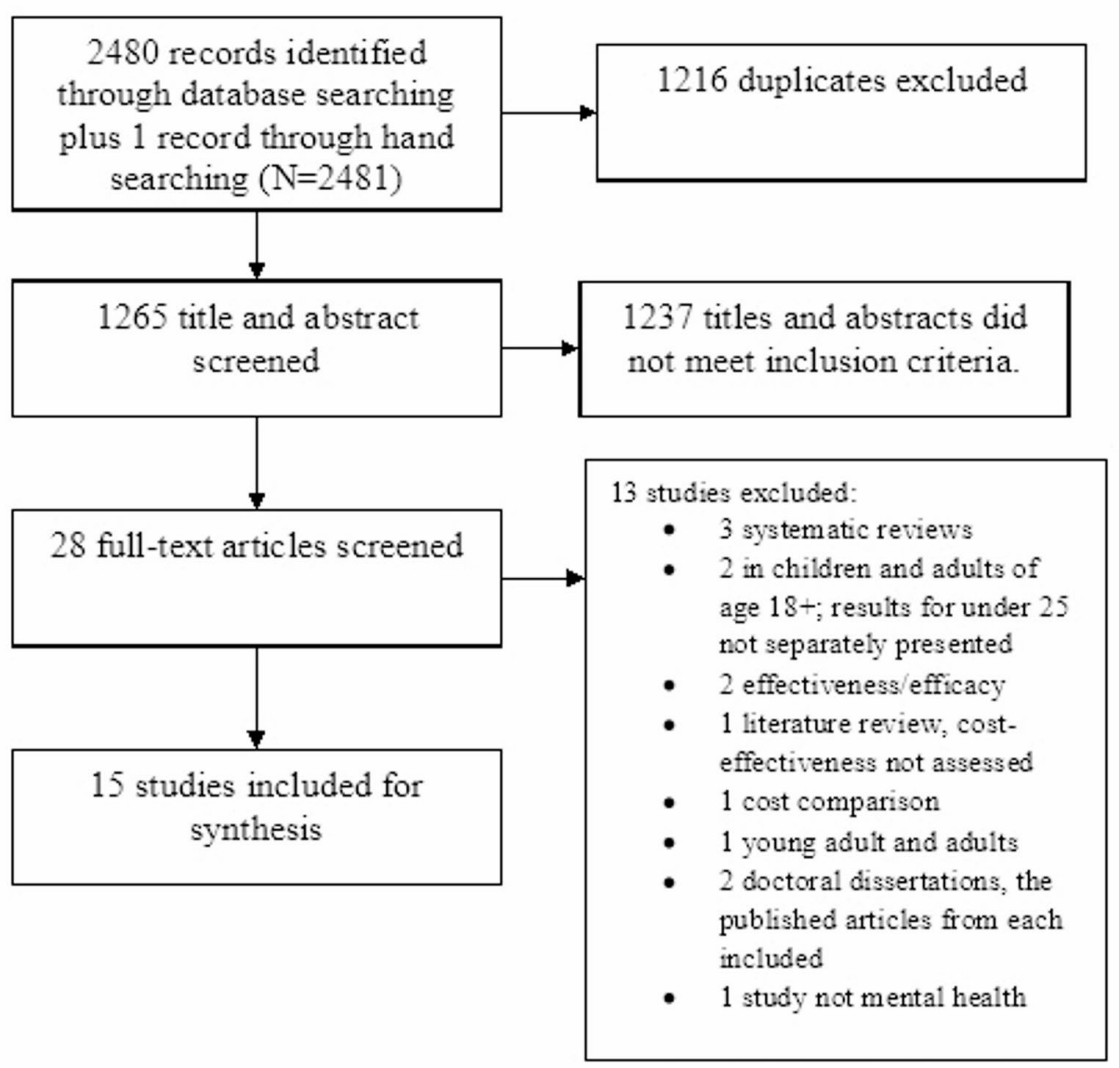
PRISMA flow diagram of study selection

The database search identified 2480 potential studies for inclusion. An additional study was retrieved through hand searching. After duplicates (1216) were removed, titles and abstracts of 1265 studies were screened, and 28 articles met the inclusion criteria for full-text screening. Full-text screening resulted in 15 studies, with moderate to high quality that were, included for the synthesis. Figure 1 depicts the process of study abstraction.

The characteristics of the studies included in this rapid review are presented in Table 2. Five of the studies were conducted in Sweden [47–49, 51, 55], four in the UK [50, 58–60]. Two studies were from Australia [53, 54]. Norway [52], Spain [56], Kenya [57], Colombia [61] had one study each. The studies were of moderate [47–53, 55–59] and high quality [54, 60, 61] (Supplementary Material 2). The mental health outcomes in the studies were anxiety [51, 54, 55, 59], depression [57, 58], obsessive compulsive disorder [49], Tourette’s syndrome (TS) and chronic tic disorder (CTD) [47, 48], distress (due to visible difference in facial or bodily appearance) [52], alcohol consumption [50], binge drinking [56], self-harm [60] and any mental illness [53, 61]. Two of the studies were model-based economic evaluations [54, 61]. The study sample sizes in trial-based studies varied between 102 and 443 and the age of participants ranged from 8- to 25-years.

The digital interventions evaluated were therapist-guided or unguided computerised (internet browser based) cognitive behaviour therapy (CBT) [49, 51, 55, 58, 59], other web-based interventions that had a CBT component [54] or CBT and social skills training [52], exposure and response prevention [47, 48] and other online interventions [50, 53, 56, 57, 60, 61]. Five studies included active comparators [47, 48, 51, 55, 57] while the rest had usual care (in-person CBT, therapist delivered internet CBT, waitlist control, or nothing) as a comparator. One of the studies [50] compared two interventions with usual care. In therapist-guided interventions, the role of the therapists was limited to answering general questions, encouraging participants, and help troubleshooting either asynchronously through text messaging or via online and/or telephone calls. Some of the trials had parallel parent interventions [51, 55, 59] to better support children.

The contents of the interventions were designed as interactive modules or chapters using a mix of presentations including text messages, narration, illustrations and/or videos. Two of the studies had an intervention with a gaming aspect [50, 59]. Some of the interventions included exercises and homework needed to be completed to proceed to successive sessions [47, 48, 51, 54]. One of the interventions [55] had two group exercise modules preceded by 12 weeks of internet CBT. Two interventions provided optional ‘add-on’ [58] or booster [52] sessions. Some of the interventions were delivered at predefined venues such as schools, health facilities, or community centres while others were completed at the participant’s home.

In terms of duration and intensity of trial-based interventions, Wasil et al. [57] had the least number of modules (three) which was completed in one session in a single school. Four studies had 5 to 8 modules [52, 54, 56, 58, 59] and another five had 10 to 14 modules (excluding booster or follow-up group exercises) [47–49, 51, 55] with one completed per week. Two studies had tools that could have been used at any time during the intervention period [50, 53, 60, 61].

One of the studies conducted CEA only [57] whereas six conducted CUA only [52–54, 58, 60, 61], the rest studies conducted both CEA and CUA. Effects of the interventions in the CEAs were measured using appropriate clinical measurements, such as the Social Anxiety Scale for Adolescents (SAS-A) [52] and Patient Health Questionnaire (PHQ) (depression) [53, 58] (Table 2) while QALYs were used to measure effects in the CUAs. Provider [52, 53, 58, 61] societal [51] and multiple perspectives [47–50, 55–57, 59, 60] were used for estimating costs and benefits in trial-based evaluations. The model-based studies also used provider [61] and societal perspective [54]. Follow-up periods varied between 2 weeks [57] and 12 months [48, 50, 58] for the trial-based studies, while the model-based evaluations modelled cost-effectiveness over 10 years [54, 61].

### Cost-effectiveness and cost-utility analyses

#### Trial-based economic evaluations

##### Societal perspective

Jolstedt et al. [51] evaluated the cost-effectiveness of internet-delivered CBT (ICBT) for anxiety in children aged 8–12 years in relation to an active comparator called internet-delivered child-directed play (ICDP) in Sweden from a societal perspective. In the ICDP, parents were instructed to play with their child in a non-directive and praising manner 2–3 times a week for about 20 min. The severity of anxiety reduced, as measured by Clinician Severity Rating (CSR) by the end of three months in both groups but more so in the ICBT group. The incremental cost (95% CI) of ICBT was 695.36 (672.96 to 717.74), the mean difference in CSR was 0.53 (0.12 to 0.87) and difference in QALYs was 0.02 (0.02 to 0.02). ICBT was not cost-effective when QALY was used as a measure of effect, thus ICUR was not reported. In CEA, ICBT was found to be cost saving and more effective with ICER (95% uncertainty interval) estimated at -US$ 2,026.36 (−2,106.11 to −1,946.60) based on remission from anxiety. The probabilities of cost-effectiveness were reported to be 80% and 100% if the WTP more for an additional effect of ICBT was zero and US$ 5,369.93, respectively.

##### Provider perspective

Le et al. [53], Wright et al. [58] and Kling et al. [52] assessed cost-effectiveness of the respective interventions from provider perspectives. The intervention in one of the studies [53], ‘Link’, was an online mental health help-seeking tool which guides users to appropriate sources of mental health information and care. After indicating the type of mental health challenge experienced, severity, and preferences for care, users were provided with information on service options, costs and online links or directories. The WTP threshold of US$ 19,755 per QALY gained was used to judge cost-effectiveness over a 3-month follow-up time. Based on both ITT and PP analyses, the intervention was dominant. The probability that ‘Link’ was cost-effective was 95% for a minimum WTP of US$ 7,047 per QALY gained compared to the usual web search strategy.

The second study [58] with a provider perspective, assessed the cost-effectiveness of a computerised CBT (CCBT), ‘Stressbusters’, compared with self-help websites for depression in adolescents in England over a 12-month follow-up time. ‘Stressbusters’ was found to be less costly and more effective resulting in negative ICUR. Based on a WTP threshold between US$ 28,694 and 43,042 per QALY, ‘Stressbusters’ had just over 65% probability of being cost-effective. The third study [52] evaluated web-based psychological intervention for psychological distress due to difference in facial and physical appearance as compared to care as usual in Norway. Cost to the control group was assumed zero due to unavailability of services for the type of distress under study. The cost for the intervention was estimated at US$ 37.00 per patient per year. The incremental effectiveness of the intervention was 0.0039 QALYs resulting in ICUR of US$ 9,418.12 per QALY. The results showed that the intervention had an 83% probability of being cost-effective based on a WTP threshold of US$ 50,400.

##### Multiple perspectives

Nine studies assessed the cost-effectiveness of digital interventions from at least two perspectives [47–50, 55–57, 59, 60]. Andrén et al. [47] and Andrén et al. [48] evaluated the same DHI at 3 months and 12 months, respectively. The DHI was an internet-delivered exposure and response prevention (ERP) intervention for Tourette’s syndrome (TS) and chronic tic disorder (CTD) compared to a therapist-supported internet-delivered education intervention in Sweden which was designed to match the ERP intervention. At 3 months, the ICER per treatment response varied between US$ 89 (health facility), and US$ 540 (health sector) while the ICUR per QALY were US$ 5,496 and US$ 37,596 based on the health facility and health sector perspectives, respectively. Depending on the perspective selected (provider, health sector, or societal), the probability that ERP was cost-effective at 3 months varied between 66% and 76% in relation to a WTP threshold of US$ 79,000 per QALY for Sweden. At 12 months, the intervention was dominant from the health sector perspective both in CEA and CUA and had 65% to 78% probability of being cost-effective from the health facility or societal perspective.

Aspvall et al. [49] and Nordh et al. [55] evaluated two separate trials conducted in Sweden to assess cost-effectiveness of therapist guided ICBT for obsessive compulsive disorder (OCD) and social anxiety disorder (SAD), respectively. The therapist-guided ICBT for OCD was evaluated against in-person CBT from health professional, health sector, and societal perspectives [49]. The CEA showed comparable effectiveness of the two therapies in terms of treatment response, but the guided ICBT was less costly than in-person CBT from all the three perspectives. When QALYs were used as a measure of effect, guided ICBT was less costly and less effective from all the three perspectives. The authors concluded that the intervention was cost-effective compared to the comparator, but ICER/ICUR and WTP threshold were not provided. In contrast to the trial on OCD, the trial on SAD had an active comparator, ISSUPORT, a child and parental online intervention that did not constitute active CBT [55]. From a societal perspective, ICBT was more effective and cost saving with ICER (95% CI) per remitter status estimated at -US$ 26,324 (−28,667 to −23,981). Using QALYs, ICBT was less effective and less costly than ISSUPORT. ICUR and WTP threshold were not provided.

Vargas-Martínez et al. [56] and Deluca et al. [50] assessed the cost-effectiveness of separate digital interventions aimed at reducing binge drinking (BD) and alcohol consumption in Spain and England, respectively. The Spanish trial was a web-based intervention, that provided information on risks of BD, preventive messages, and benefits of not drinking. The intervention was evaluated against waitlist control, from the Spanish Health System (NHS) and societal perspectives. Using a WTP threshold of US$ 32,012 to 36,585 per QALY, the intervention was dominant from the societal perspective and cost-effective from the NHS perspective with ICER and ICUR estimated at US$ 31.64 per BD occasions and US$ 135.15 per QALY, respectively. A deterministic sensitivity analysis showed that the intervention could be dominant even in a worst-case scenario from the societal perspective. The worst-case scenario was determined by the minimum value of the 95% confidence intervals of BD occasions avoided or number of QALYs gained. Additional sensitivity analyses demonstrated differences in cost-effectiveness by gender and age. When BD occasions averted was used as the measure of effect, the intervention was dominant for girls from both the NHS and societal perspectives but not cost-effective for boys. Similarly, based on BD occasions averted, the intervention was dominant for older adolescents (aged 17 or greater) from the NHS perspective. When QALY was used, the intervention was dominant for younger participants (aged less than 17) from the societal perspective.

Deluca et al. [50] conducted a cost-effectiveness analysis comparing two interventions, personalised feedback plus smartphone (eBI) and personalised feedback and brief advice (PFBA) with ‘screening only’, in low-risk adolescents attending emergency department (ED) in England. The two interventions included information on risks of alcohol consumption and provided personalized feedback, the difference between the two interventions being an added gaming aspect in the eBI using smartphone or PC based app. Considering societal and national health services-personal social services (NHS-PSS) perspectives, the eBI intervention was not cost-effective compared to ‘screening only’ when evaluated against a WTP threshold of US$ 28,777 to 43,165 per QALY. In a probabilistic sensitivity analysis, eBI had only 9% probability of being cost-effective based on either perspective.

Creswell et al. [59] evaluated the cost-effectiveness of an Online Support and Intervention (OSI) supported by therapist (OSI + TS) compared to treatment as usual in children with anxiety problems in the UK. The intervention was a parent-led ‘digitally augmented’ CBT supported by therapist with optional gaming aspect. Cost-effectiveness was assessed over 24 weeks from the NHS-PSS perspective. Based on an ITT, the incremental cost (95% CI) was -US$ 142.86 (−412.68 to 126.95) and the incremental QALYs (95% CI) were − 0.007 (−0.015 to 0.002) based on the UK adult value set and − 0.002 (−0.017 to 0.012) based on the Australian (AU) adolescent value set. Consequently, ICURs per QALY were estimated at US$ 21,433 and US$ 63,045 based on the UK adult value set and the AU adolescent value set, respectively. In reference to a WTP threshold of US$ 25,615 to 38,422, the probabilities of cost-effectiveness were 30% and 24% (UK adult value set) and 60% and 53% (AU adolescent value set). Another trial from the UK assessed a self-help smartphone app (Usual Care plus BlueIce (UC + BI)) for self-harm compared to usual care [60]. The incremental costs (95% CI) were -US$ 394 (−1,455 to 666) at 12 weeks and -US$ 1,139 (−2,807 to 528) at 6 months while the incremental QALYs were 0.003 (−0.005 to 0.017) and 0.004 (−0.001 to 0.010), respectively. Net monetary benefit was US$ 520.55 (12-weeks) and US$ 1,234.13 (6-months) and the probability of cost-effectiveness was 75% (12-weeks) and 70% (6-months) with respect to the WTP threshold of US$ 28,777.

The last study in this group, and the only study from Africa, examined whether an online single session self-help intervention for depression was cost-effective relative to an active comparator focused on study skills in school children in Kenya [57]. Both interventions were developed specifically for adolescents in Kenya. School, researcher, and societal perspectives were considered to compare effectiveness per cost ratio since costs were assumed to be the same. The results showed a 0.5 reduction in PHQ-9 per US dollar spent in the intervention compared to no improvement in the comparator.

#### Model based economic evaluations

Two of the studies included in this review assessed cost-effectiveness of DHIs based on simulation models over a time horizon of 10 years. Lee et al. [54] used a Markov model to investigate the cost-effectiveness and return on investment of an internet-based intervention (MoodGYM) in comparison to ‘no intervention’ for anxiety from a societal perspective. MoodGYM included a CBT content and had been widely introduced across schools in Australia. The incremental QALYs and costs over 10 years were estimated at 842 (289 to 1,681) and -US$ 11.28 million (−38.2 million to −1.51 million), respectively. In reference to a WTP threshold of US$ 35,261 per QALY, the ICUR was found to be dominant demonstrating that MoodGYM had both improved clinical effectiveness and produced net cost-savings. The return on investment for US$ 0.89 (Aus$ 1) investment was estimated at US$ 16.7 (Aus$ 3.06 in 2016). Univariate sensitivity analyses were conducted using different scenarios including (but not limited to) modelling only for younger participants (11-year-olds) or older participants (17-year-olds) or changing subscription cost of the interventions. Overall, the sensitivity analyses showed that results were robust except for the changes in subscription from school to students which resulted in the intervention being no more cost-effective.

In comparison, Natsky et al. [61] used a systems dynamics model to assess cost-effectiveness of nine mental health interventions in relation to business as usual (BAU) from a provider perspective in Colombia. Out of these interventions, two were suicide helplines and self-help mental health services delivered online. The incremental costs (95% CI) were estimated at -US$ 1,930,912.65 (−2,427,960.63 to −1,499,286.71) for online mental health services and US$ 3,433,515.84 (2,729,607.69 to 4,153,919.45) for suicide helplines. The incremental QALYs were 10,164 (8,549 to 11,870) and 579 (31 to 1,070), respectively. Using a cost-effectiveness threshold of US$ 4,890 per QALY, the online mental health service was dominant, with an incremental net monetary benefit (INMB) of US$ 55.13 (45.59 to 63.62). The ICUR for suicide helplines was US$ 5,932 per QALY indicating that the suicide helpline was not cost-effective over the 10-year time horizon.

## Discussion

The aim of this rapid review was to assess the cost-effectiveness of DHIs for supporting poor mental health in children and young people (CYP) up to the age of 25 years. Most of the evaluated interventions were digital versions of conventional therapies such as CBT, or they included some components of CBT. Social skills training and exposure and response prevention were also among the interventions. Generally, the synthesis suggests that there is evidence to support the use of DHIs for child and adolescent mental health problems, and that DHIs can be cost-effective. The included studies, except two, reported interventions to be cost-effective based on, at least, one outcome measure or the probability of cost-effectiveness was greater than 60%. In two of trial-based studies [50, 59], the probabilities of cost-effectiveness were less 60%. The two model-based studies [54, 61] and three trial-based studies [48, 53, 56] found the interventions to be dominant from at least one perspective. While most of the results indicate cost-effectiveness of the DHIs, this needs to be interpreted with caution due to differences in health systems and WTP thresholds across the countries, measures of effect (e.g. response to treatment or QALYs), costing perspective (e.g. provider or societal), and assumptions of characteristics and number of users of the technologies.

Differences in health care systems and other local contextual factors where interventions were carried out influences cost-effectiveness [71, 72]. All the studies included in this review, were conducted in high-income countries (HICs) (Australia, Norway, Sweden, Spain and United Kingdom) except two, which were from Colombia and Kenya [57, 61]. The HICs included in this review share similarities in their health systems being predominantly publicly funded albeit with some differences especially between Nordic and other OECD countries [73]. For instance, per capita health expenditure in 2022 PPP US$ was 7,771 (Norway), 6,438 (Sweden), 6,372 (Australia), 5,493 (UK), 4,432 (Spain), 1,640 (Colombia) and 94.67 (Kenya – domestic public funding) [73, 74]. Resource availability is a key factor in policy makers’ decisions of what services to provide [75]. Cost-effectiveness analyses are widely used in publicly funded system to inform decisions of coverage of treatments to maximise value for money from public resources while its use is limited in private and mixed health care systems with less public funding and significant private payments [76]. Furthermore, cost-effectiveness results can vary within countries: an intervention which is cost-effective in one context (e.g. urban area) may not necessarily be the same in another context (e.g. rural area) [72]. Contextual demand-side (e.g., perceptions of mental illness, healthcare seeking behaviours) and supply-side (e.g., healthcare policies, structures, and access to care) factors [77] that influence any health intervention also have a bearing on DHIs including perception of usability of digital tools for mental health therapy [50, 55, 78]. Cost-effectiveness of interventions also depend on a country’s WTP threshold that represents the maximum amount of money a payer (country) is willing to pay per effect [46]. In this review, WTP thresholds in US$ for the study periods varied between US$ 4,890 for Colombia [61] to US$ 79,000 for Sweden [47, 48]. This indicates, for instance, that an intervention that was found to be cost-effective the Sweden using US$ 79,000 threshold will not necessarily be cost-effective in reference to the Columbian WTP threshold.

Sub-group analysis sheds light on differences in cost-effectiveness between different sub-groups of participants. This was shown by sensitivity analyses in two of the included studies by gender and age [54, 56]. Vargas-Martínez et al. [56] found that the trial-based intervention that aimed at reducing binge drinking was more effective in girls while Lee et al. [54], in model-based evaluation, showed that the intervention comparably cost-effective in separate modelling for young (age 11 years) and older (17 years) participants [56]. It is important to assess whether cost-effective varies across different groups of patients or setting and make relevant adaptations to improve effectiveness [79]. Most of the studies included in this review did not detail specific contextual factors in the study settings that might have affected treatment responses, and in what contexts the findings would be generalisable. Furthermore, some studies recruited study participants through online advertising and social media [49, 51, 53], which introduces selection bias. While recruitment using social media is efficient and cost-effective [80], it is not clear if (how) the recruitment strategies were reflected in the treatment outcomes. For example, differences in outcomes have been shown between participants recruited through online strategies or medical registries for distress related to cancer [81]. It has also been argued that although some DHIs have been effective, they have had limited impact on the broader healthcare system [38]. Therefore, there is a need for a greater implementation detail of DHIs (e.g., perceptions of users, participation of users in design of DHIs, level of users’ engagement, level of therapist involvement, change in relational power between therapists and users, and regulatory complexities) in future studies to enable a more comprehensive assessment of the implementation components that are key to cost-effectiveness assessments [82–84]. The international Collaboration On Maximising the impact of E-Therapy and Serious Gaming (COMETS) suggests user-centred design, greater individualisation, improved adherence, increased intersectoral collaboration, and rapid testing and implementation to increase the effect of DHIs on improving mental health [85].

Short follow-up periods in trial-based studies means that longer-term implications of these interventions are unknown. However, the model-based studies complemented the trial-based studies by assessing cost-effectiveness over 10 years [54, 61] although the two approaches are not directly comparable. One of the key challenges in longitudinal studies of digital interventions is that software, or the operating platforms can become obsolete as technology advances. The average lifespan of a software application is 6–8 years, and mobile apps have an even shorter lifespan due to upgrades of the operating system and hardware. Mobile apps that are published to a store and not maintained may require a complete code rewrite every one to three years. Over a 10-year period, the digital interventions studied could become obsolete and/or cease to function [86]. Furthermore, DHIs that incorporate Artificial Intelligence (AI) (e.g. chatbots), which interact with users through different models (e.g. machine learning), have been increasing over recent years making traditional DHI tools and apps quickly outdated [87, 88].

Definitive conclusions regarding the cost-effectiveness of DHIs for child and adolescent mental health problems are further limited by the heterogeneity in the DHIs and therapeutic approaches that were evaluated by the included studies. Evaluation of the same therapeutic approach (e.g. CBT) with different modes of delivery (e.g., face-to-face versus digital) may confirm whether the mode of delivery is cost-effective (e.g. Aspvall et al. [49]). Comparing one therapeutic approach (e.g., CBT) delivered digitally with another therapeutic approach (e.g., DBT) delivered face-to-face (or a mix of modalities) does not necessarily indicate whether cost-effectiveness of the intervention is due to the therapeutic approach itself, or due to the mode of delivery. Such variation in controls in RCTs of DHIs may bias treatment outcomes of the interventions [89]. Therefore, given that various therapeutic approaches were evaluated in the included studies, the cost-effectiveness evidence of this review needs to be interpreted accordingly. This also means that ‘head-to-head’ evaluations of digital and non-digital interventions of the same therapeutic kind are needed to draw definitive conclusions about the effectiveness of digital modalities of specific therapeutic approaches. Considering, the issues discussed above, the conclusions of cost-effectiveness should be interpreted with care and take account of methodological choices of studies. More high-quality evidence with comparable methodological approaches is needed to inform implementation decision as discussed above.

Rapid reviews are justified when evidence is needed quickly [90, 91]. However, rapid reviews have several limitations. There are several compromises compared with standard systematic reviews [92]. First, in the context of the current review, only a few databases were searched, and a simple broad search strategy was employed. Second, the review included trial-based, and model-based economic evaluation which are not directly comparable. Trial-based economic evaluations are conducted using data from trial participants while the input parameters for model-based intervention are gathered from various sources, simulated for hypothetical participant and valid under certain assumptions that are made. Third, the economic evaluations are conducted under different health systems and results may not be generalisable. Fourth, one reviewer screened records and extracted data with a second reviewer screening 10% of the studies. This may have affected the precision of study selection [93]. Fifth, by conducting the review in a short duration of time, publication selection bias may have occurred. However, review of three published systematic reviews [20, 35, 36] during the screening stage indicated that important studies may have not been missed. Sixth, quality assessment was limited to certain aspects of quality (Supplementary Material 2) which may have introduced bias in the assessments. Furthermore, some limitations of the studies included in the review limit generalisability, including small sample sizes, limited assessment of heterogeneity in cost-effectiveness, and unclear contextual factors (such as digital access issues, and local policies). Finally, this rapid review was not prospectively registered, which might have resulted in bias pertaining to method of synthesis and/or duplicate reviews.

This rapid review provides some evidence to suggest that digital interventions have the potential to expand healthcare coverage for CYP with mental health problems thus addressing the treatment gap that currently exists. Our review included both standalone and therapist-guided interventions; however, it was not always possible to accurately determine the level of therapist involvement. More robust and long-term studies, with varying levels of therapist involvement, are needed to strengthen the evidence base of the cost-effectiveness of DHI for supporting mental health of CYP. Determining the minimal level of in-person support needed without compromising outcomes is crucial, given the ever-increasing demand for mental health services coupled with shortages of professional therapists, and other access issues. Digital interventions could serve as the first contact with the health care system for mild to moderate mental health problems, increasing capacity within clinical services and children and adolescents’ access to therapies. However, any developments in this regard should consider individuals who might be excluded (for example, due to digital poverty), and specifically address the associated structural disadvantages. Synthesis of evidence on cost-effectiveness of DHIs would be effective if studies follow standardised reporting of outcomes (e.g., effective ontologies for mental health outcomes in digital mental health), methods (e.g., co-design, intensity of use, fidelity of intervention implementation), and challenges (e.g. software updates, technical issues) [94, 95]. Furthermore, cost-effectiveness must be evaluated alongside ethical considerations, including the implications of shifts in responsibility from clinicians to users, potential task and labour replacements, and the broader societal impacts. Ensuring appropriate professional guidance and regulation is therefore essential to deliver safe and high-quality, as well as cost-effective care [89, 96].

## Supplementary Information

Below is the link to the electronic supplementary material.

ESM 1 (DOCX 29.0 KB)

## Data Availability

Not applicable.

## Footnotes

1 CADTH. Economic Evaluations & Models - MEDLINE: Ottawa: CADTH; 2023 [Available from: https://searchfilters.cadth.ca/link/16].

## References

[1] Global Burden of Disease Collaborative Network ‘Global Burden of Disease Study 2021 Results’, Seattle, United States

[2] Scott J, Mihalopoulos C, Erskine H, Roberts J, Rahman A (2016) ‘Childhood Mental and Developmental Disorders’, in Mental, Neurological, and Substance Use Disorders: Disease Control Priorities, 3rd ed., V. Patel, D. Chisholm, T. Dua, R. Laxminarayan, and M. Medina-Mora, Eds., The International Bank for Reconstruction and Development / The World Bank, ch. 8

[3] Doering S, Lichtenstein P, Gillberg C, Kuja-Halkola R, Lundström S (2021) Internalizing and neurodevelopmental problems in young people: educational outcomes in a large population-based cohort of twins. Psychiatry Res 298:113794. 10.1016/j.psychres.2021.113794 33596506 10.1016/j.psychres.2021.113794

[4] Goodwin RD et al (2009) Do mental health problems in childhood predict chronic physical conditions among males in early adulthood? Evidence from a community-based prospective study. Psychol Med 39(2):301–311. 10.1017/S0033291708003504 10.1017/S0033291708003504 18507873

[5] Alonso J et al (2014) Association between mental disorders and subsequent adult onset asthma. J Psychiatr Res 59:179–188. 10.1016/j.jpsychires.2014.09.007 25263276 10.1016/j.jpsychires.2014.09.007 PMC5120389

[6] McLeod JD, Fettes DL (2007) Trajectories of failure: the educational careers of children with mental health problems. Am J Sociol 113(3):653–701. 10.1086/521849 10.1086/521849 PMC2766187 19855855

[7] Mojtabai R, Stuart EA, Hwang I, Eaton WW, Sampson N, Kessler RC (2015) Long-term effects of mental disorders on educational attainment in the National Comorbidity Survey ten-year follow-up. Soc Psychiatry Psychiatr Epidemiol 50(10):1577–1591. 10.1007/s00127-015-1083-5 26082040 10.1007/s00127-015-1083-5 PMC4964966

[8] Goodman A, Joyce R, Smith JP (2011) The long shadow cast by childhood physical and mental problems on adult life. Proc Natl Acad Sci U S A 108(15):6032–6037. 10.1073/pnas.1016970108 21444801 10.1073/pnas.1016970108 PMC3076863

[9] Copeland WE, Wolke D, Shanahan L, Costello EJ (2015) Adult functional outcomes of common childhood psychiatric problems. JAMA Psychiatr 72(9):892. 10.1001/jamapsychiatry.2015.0730 10.1001/jamapsychiatry.2015.0730 PMC4706225 26176785

[10] Kessler RC et al (2007) ‘Lifetime prevalence and age-of-onset distributions of mental disorders in the World Health Organization’s World Mental Health Survey Initiative.’, World Psychiatry 6(3):168–76 PMC2174588 18188442

[11] Mulraney M et al (2021) A systematic review of the persistence of childhood mental health problems into adulthood. Neurosci Biobehav Rev 129:182–205. 10.1016/j.neubiorev.2021.07.030 34363845 10.1016/j.neubiorev.2021.07.030

[12] Gulliver A, Griffiths KM, Christensen H (2010) Perceived barriers and facilitators to mental health help-seeking in young people: a systematic review. BMC Psychiatry 10(1):113. 10.1186/1471-244X-10-113 21192795 10.1186/1471-244X-10-113 PMC3022639

[13] Salaheddin K, Mason B (2016) Identifying barriers to mental health help-seeking among young adults in the UK: a cross-sectional survey. Br J Gen Pract 66(651):e686–e692. 10.3399/bjgp16X687313 27688518 10.3399/bjgp16X687313 PMC5033305

[14] Radez J, Reardon T, Creswell C, Lawrence PJ, Evdoka-Burton G, Waite P (2021) Why do children and adolescents (not) seek and access professional help for their mental health problems? A systematic review of quantitative and qualitative studies. Eur Child Adolesc Psychiatry 30(2):183–211. 10.1007/s00787-019-01469-4 31965309 10.1007/s00787-019-01469-4 PMC7932953

[15] World Health Organization (2022) ‘World mental health report: transforming mental health for all ’, Geneva

[16] Byrne A, Barber R, Lim CH (2021) Impact of the < scp > COVID -19 pandemic – a mental health service perspective. Prog Neurol Psychiatry 25(2):27. 10.1002/pnp.708

[17] Gupta N, Dhamija S, Patil J, Chaudhari B (2021) Impact of COVID-19 pandemic on healthcare workers. Ind Psychiatry J 30(Suppl 1):S282–S284. 10.4103/0972-6748.328830 34908710 10.4103/0972-6748.328830 PMC8611576

[18] World Health Organisation (2021) ‘Mental Health Atlas 2020’, Geneva

[19] Dua T et al (2011) Evidence-based guidelines for mental, neurological, and substance use disorders in low- and middle-income countries: summary of WHO recommendations. PLoS Med 8(11):e1001122. 10.1371/journal.pmed.1001122 22110406 10.1371/journal.pmed.1001122 PMC3217030

[20] Hollis C et al (2017) Annual research review: digital health interventions for children and young people with mental health problems – a systematic and meta-review. J Child Psychol Psychiatry 58(4):474–503. 10.1111/jcpp.12663 27943285 10.1111/jcpp.12663

[21] Andersson G, Titov N (2014) Advantages and limitations of internet-based interventions for common mental disorders. World Psychiatry 13(1):4–11. 10.1002/wps.20083 24497236 10.1002/wps.20083 PMC3918007

[22] Bergin AD et al (2020) Preventive digital mental health interventions for children and young people: a review of the design and reporting of research. NPJ Digit Med 3(1):133. 10.1038/s41746-020-00339-7 33083568 10.1038/s41746-020-00339-7 PMC7562906

[23] Egan JE et al (2021) Feasibility of a web-accessible game-based intervention aimed at improving help seeking and coping among sexual and gender minority youth: results from a randomized controlled trial. J Adolesc Health 69(4):604–614. 10.1016/j.jadohealth.2021.03.027 34140199 10.1016/j.jadohealth.2021.03.027 PMC8494066

[24] Douma M et al (2021) Online psychosocial group intervention for adolescents with a chronic illness: a randomized controlled trial. Internet Interv 26:100447. 10.1016/j.invent.2021.100447 34485096 10.1016/j.invent.2021.100447 PMC8405893

[25] Bohleber L, Crameri A, Eich-Stierli B, Telesko R, von Wyl A (2016) Can we foster a culture of peer support and promote mental health in adolescence using a web-based app? A control group study. JMIR Ment Health 3(3):e45. 10.2196/mental.5597 27663691 10.2196/mental.5597 PMC5074648

[26] Abd-alrazaq A et al (2022) The effectiveness of serious games in alleviating anxiety: systematic review and meta-analysis. JMIR Serious Games 10(1):e29137. 10.2196/29137 35156932 10.2196/29137 PMC8887639

[27] Lattie EG, Adkins EC, Winquist N, Stiles-Shields C, Wafford QE, Graham AK (2019) Digital mental health interventions for depression, anxiety, and enhancement of psychological well-being among college students: systematic review. J Med Internet Res 21(7):e12869. 10.2196/12869 31333198 10.2196/12869 PMC6681642

[28] Liverpool S et al (2020) Engaging children and young people in digital mental health interventions: systematic review of modes of delivery, facilitators, and barriers. J Med Internet Res 22(6):e16317. 10.2196/16317 32442160 10.2196/16317 PMC7381028

[29] Mittmann G, Barnard A, Krammer I, Martins D, Dias J (2022) ‘LINA - A Social Augmented Reality Game around Mental Health, Supporting Real-world Connection and Sense of Belonging for Early Adolescents’, Proc ACM Hum Comput Interact vol. 6, no. CHI PLAY, pp. 1–21. 10.1145/3549505

[30] Hugh-Jones S et al (2022) Adolescents accept digital mental health support in schools: a co-design and feasibility study of a school-based app for UK adolescents. Mental Health & Prevention 27:200241. 10.1016/j.mhp.2022.200241

[31] Hugh-Jones S, Ulor M, Nugent T, Walshe S, Kirk M (2023) The potential of virtual reality to support adolescent mental well-being in schools: a UK co-design and proof-of-concept study. Mental Health & Prevention 30:200265. 10.1016/j.mhp.2023.200265

[32] Kruzan KP, Biernesser C, Hoffmann JA, Meyerhoff J (2024) Digital interventions for adolescents and young adults experiencing self-injurious thoughts and behaviors. Curr Treat Options Psychiatry 11(2):76–89. 10.1007/s40501-024-00318-9 39525358 10.1007/s40501-024-00318-9 PMC11548831

[33] Wright M, Reitegger F, Cela H, Papst A, Gasteiger-Klicpera B (2023) Interventions with digital tools for mental health promotion among 11–18 year olds: a systematic review and meta-analysis. J Youth Adolesc 52(4):754–779. 10.1007/s10964-023-01735-4 36754917 10.1007/s10964-023-01735-4 PMC9907880

[34] Lehtimaki S, Martic J, Wahl B, Foster KT, Schwalbe N (2021) Evidence on digital mental health interventions for adolescents and young people: systematic overview. JMIR Ment Health 8(4):e25847. 10.2196/25847 33913817 10.2196/25847 PMC8120421

[35] Rohrbach PJ et al (2023) Cost-effectiveness of internet interventions compared with treatment as usual for people with mental disorders: systematic review and meta-analysis of randomized controlled trials. J Med Internet Res 25:e38204. 10.2196/38204 36602854 10.2196/38204 PMC9893732

[36] Kählke F, Buntrock C, Smit F, Ebert DD (2022) Systematic review of economic evaluations for internet- and mobile-based interventions for mental health problems. NPJ Digit Med 5(1):175. 10.1038/s41746-022-00702-w 36424463 10.1038/s41746-022-00702-w PMC9686241

[37] Grist R, Porter J, Stallard P (2017) Mental health mobile apps for preadolescents and adolescents: a systematic review. J Med Internet Res 19(5):e176. 10.2196/jmir.7332 28546138 10.2196/jmir.7332 PMC5465380

[38] Wilkinson T, Wang M, Friedman J, Prestidge M (2023) ‘A framework for the economic evaluation of digital health interventions’, Washington D.C 10.1093/oodh/oqae028 PMC11936328 40230550

[39] Gomes M, Murray E, Raftery J (2022) Economic evaluation of digital health interventions: methodological issues and recommendations for practice. Pharmacoeconomics 40(4):367–378. 10.1007/s40273-022-01130-0 35132606 10.1007/s40273-022-01130-0 PMC8821841

[40] Roberts RE, Roberts CR, Xing Y (2007) Comorbidity of substance use disorders and other psychiatric disorders among adolescents: evidence from an epidemiologic survey. Drug Alcohol Depend 88:S4–S13. 10.1016/j.drugalcdep.2006.12.010 17275212 10.1016/j.drugalcdep.2006.12.010 PMC1935413

[41] Mann AP et al (2014) Factors associated with substance use in adolescents with eating disorders. J Adolesc Health 55(2):182–187. 10.1016/j.jadohealth.2014.01.015 24656448 10.1016/j.jadohealth.2014.01.015 PMC4108497

[42] Moher D et al (2015) Preferred reporting items for systematic review and meta-analysis protocols (PRISMA-P) 2015 statement. Syst Rev 4(1):1. 10.1186/2046-4053-4-1 25554246 10.1186/2046-4053-4-1 PMC4320440

[43] Ayiku L et al (2021) The NICE MEDLINE and Embase (Ovid) health apps search filters: development of validated filters to retrieve evidence about health apps. Int J Technol Assess Health Care 37(1):e16. 10.1017/S026646232000080X 10.1017/S026646232000080X 33107420

[44] Bear HA et al (2022) Determination of markers of successful implementation of mental health apps for young people: systematic review. J Med Internet Res 24(11):e40347. 10.2196/40347 36350704 10.2196/40347 PMC9685513

[45] Centre for reviews and dissemination (2006) Systematic reviews: crd’s guidance for undertaking reviews in health care. ’, York

[46] Drummond MF, Sculpher MJ, Claxton K, Stoddart GL, Torrance GW (2015) Methods for the economic evaluation of health care programmes, 3rd edn. Oxford University Press

[47] Andrén P et al (2022) Therapist-supported internet-delivered exposure and response prevention for children and adolescents with Tourette syndrome. JAMA Netw Open 5(8):e2225614. 10.1001/jamanetworkopen.2022.25614 35969401 10.1001/jamanetworkopen.2022.25614 PMC9379743

[48] Andrén P et al (2024) Internet-delivered exposure and response prevention for pediatric Tourette syndrome. JAMA Netw Open 7(5):e248468. 10.1001/jamanetworkopen.2024.8468 38700867 10.1001/jamanetworkopen.2024.8468 PMC11069081

[49] Aspvall K et al (2021) Cost-effectiveness of internet-delivered vs in-person cognitive behavioral therapy for children and adolescents with obsessive-compulsive disorder. JAMA Netw Open 4(7):e2118516. 10.1001/jamanetworkopen.2021.18516 34328501 10.1001/jamanetworkopen.2021.18516 PMC8325072

[50] Deluca P et al (2021) Brief interventions to prevent excessive alcohol use in adolescents at low-risk presenting to Emergency Departments: three-arm, randomised trial of effectiveness and cost-effectiveness. Int J Drug Policy 93:103113. 10.1016/j.drugpo.2021.103113 33487528 10.1016/j.drugpo.2021.103113 PMC8261826

[51] Jolstedt M et al (2018) Efficacy and cost-effectiveness of therapist-guided internet cognitive behavioural therapy for paediatric anxiety disorders: a single-centre, single-blind, randomised controlled trial. Lancet Child Adolesc Health 2(11):792–801. 10.1016/S2352-4642(18)30275-X 30241993 10.1016/S2352-4642(18)30275-X

[52] Kling J, Asphaug L, Feragen KB (2023) Cost-effectiveness analysis of a psychosocial web‐based intervention for adolescents distressed by a visible difference: results from a randomized controlled trial in Norway. Scand J Psychol 64(3):268–277. 10.1111/sjop.12885 36367227 10.1111/sjop.12885

[53] Le LK-D, Sanci L, Chatterton M. Lou, Kauer S, Buhagiar K, Mihalopoulos C (2019) The cost-effectiveness of an internet intervention to facilitate mental health help-seeking by young adults: randomized controlled trial. J Med Internet Res 21(7):e13065. 10.2196/13065 31333199 10.2196/13065 PMC6681639

[54] Lee YY, Le LK-D, Lal A, Engel L, Mihalopoulos C (2021) The cost-effectiveness of delivering an e-health intervention, MoodGYM, to prevent anxiety disorders among Australian adolescents: a model-based economic evaluation. Mental Health & Prevention 24:200210. 10.1016/j.mhp.2021.200210

[55] Nordh M et al (2021) Therapist-guided internet-delivered cognitive behavioral therapy vs internet-delivered supportive therapy for children and adolescents with social anxiety disorder. JAMA Psychiatr 78(7):705. 10.1001/jamapsychiatry.2021.0469 10.1001/jamapsychiatry.2021.0469 PMC8117054 33978699

[56] Vargas-Martínez AM, Lima‐Serrano M, Trapero‐Bertran M (2023) Cost‐effectiveness and cost‐utility analyses of a web‐based computer‐tailored intervention for prevention of binge drinking among Spanish adolescents. Alcohol Clin Exp Res 47(2):319–335. 10.1111/acer.14990 10.1111/acer.14990 36811462

[57] Wasil AR (2021) Economic evaluation of an online single-session intervention for depression in Kenyan adolescents. J Consult Clin Psychol 89(8):657–667. 10.1037/ccp0000669 34472893 10.1037/ccp0000669

[58] Wright B, Tindall L, Hargate R, Allgar V, Trépel D, Ali S (2020) Computerised cognitive–behavioural therapy for depression in adolescents: 12-month outcomes of a UK randomised controlled trial pilot study. BJPsych Open 6(1):e5. 10.1192/bjo.2019.91 10.1192/bjo.2019.91 PMC7001485 31829300

[59] Creswell C et al (2024) Digitally augmented, parent-led CBT versus treatment as usual for child anxiety problems in child mental health services in England and Northern Ireland: a pragmatic, non-inferiority, clinical effectiveness and cost-effectiveness randomised controlled trial. Lancet Psychiatr 11(3):193–209. 10.1016/S2215-0366(23)00429-7 10.1016/S2215-0366(23)00429-7 38335987

[60] Morrish N et al (2024) Cost-effectiveness of adding a smartphone app (BlueIce) to the mental health care of adolescents who repeatedly self-harm. Psychiatry Res 342:116186. 10.1016/j.psychres.2024.116186 39293280 10.1016/j.psychres.2024.116186

[61] Natsky AN (2025) Economic evaluation of 9 intersectoral strategies to improve youth mental health and alleviate financial burden in Colombia using system dynamics modeling. Value Health 28(3):389–398. 10.1016/j.jval.2024.11.004 39613257 10.1016/j.jval.2024.11.004

[62] Bertram M et al (2016) Cost–effectiveness thresholds: pros and cons. Bull World Health Organ 94(12):925–930. 10.2471/BLT.15.164418 27994285 10.2471/BLT.15.164418 PMC5153921

[63] Sittimart M et al (2024) An overview of the perspectives used in health economic evaluations. Cost Eff Resour Alloc 22(1):41. 10.1186/s12962-024-00552-1 38741138 10.1186/s12962-024-00552-1 PMC11092188

[64] Shemilt I, James T, Marcello M (2010) A web-based tool for adjusting costs to a specific target currency and price year. Evid Policy 6(1):51–59. 10.1332/174426410X482999

[65] Boodhna T, Hendrich J (2017) Shouldn’t NICE cost-effectiveness thresholds be changing with the times? Value Health 20(9):A704

[66] Menzel PT (2021) How should willingness-to-pay values of quality-adjusted life-years be updated and according to whom? AMA J Ethics 23(8):601–606 10.1001/amajethics.2021.601 34459726

[67] Tripepi G, Chesnaye NC, Dekker FW, Zoccali C, Jager KJ (2020) Intention to treat and per protocol analysis in clinical trials. Nephrology 25(7):513–517. 10.1111/nep.13709 32147926 10.1111/nep.13709

[68] Evers S, Goossens M, de Vet H, van Tulder M, Ament A (2005) Criteria list for assessment of methodological quality of economic evaluations: consensus on health economic criteria. Int J Technol Assess Health Care 21(2):240–245. 10.1017/S0266462305050324 15921065

[69] Garritty C et al (2021) Cochrane rapid reviews methods group offers evidence-informed guidance to conduct rapid reviews. J Clin Epidemiol 130:13–22. 10.1016/j.jclinepi.2020.10.007 33068715 10.1016/j.jclinepi.2020.10.007 PMC7557165

[70] Husereau D et al (2022) Consolidated health economic evaluation reporting standards 2022 (CHEERS 2022) statement: updated reporting guidance for health economic evaluations. BMC Med 20(1):23. 10.1186/s12916-021-02204-0 35022047 10.1186/s12916-021-02204-0 PMC8753858

[71] Thomas Craig KJ (2021) Systematic review of context-aware digital behavior change interventions to improve health. Transl Behav Med 11(5):1037–1048. 10.1093/tbm/ibaa099 33085767 10.1093/tbm/ibaa099 PMC8158169

[72] Whitten PS (2002) Systematic review of cost effectiveness studies of telemedicine interventions. BMJ 324(7351):1434–1437. 10.1136/bmj.324.7351.1434 12065269 10.1136/bmj.324.7351.1434 PMC115857

[73] OECD (2023) ‘Health at a Glance 2023: OECD Indicators’, Paris

[74] World Bank (2025) ‘Domestic general government health expenditure per capita, PPP (current international $) - Kenya’, Accessed June 11, 2025

[75] WHO (2010) ‘The World Health Report: Health Systems Financing: the path to universal Coverage.’, Geneva 10.2471/BLT.10.078741 PMC2878164 20539847

[76] Thomas R, Chalkidou K (2016) Cost–effectiveness analysis. In J. Cylus, I. Papanicolas, & P. C. Smith, editors, Health system efficiency: how to make measurement matter for policy and management. Health Policy Series No. 46, pp. 115-134. Copenhagen:European Observatory on Health Systems and Policies 28783269

[77] Haenssgen MJ et al (2019) How context can impact clinical trials: a multi-country qualitative case study comparison of diagnostic biomarker test interventions. Trials 20(1):111. 10.1186/s13063-019-3215-9 30736818 10.1186/s13063-019-3215-9 PMC6368827

[78] Phang YS, Heaukulani C, Martanto W, Morris R, Tong MM, Ho R (2023) Perceptions of a digital mental health platform among participants with depressive disorder, anxiety disorder, and other clinically diagnosed mental disorders in Singapore: usability and acceptability study. JMIR Hum Factors 10:e42167. 10.2196/42167 36989020 10.2196/42167 PMC10132018

[79] Abi Ramia J et al (2018) Community cognitive interviewing to inform local adaptations of an e-mental health intervention in Lebanon. Glob Ment Health 5:e39. 10.1017/gmh.2018.29 10.1017/gmh.2018.29 PMC6315334 30637112

[80] Darko EM, Kleib M, Olson J (2022) Social media use for research participant recruitment: integrative literature review. J Med Internet Res 24(8):e38015. 10.2196/38015 35925655 10.2196/38015 PMC9389385

[81] Owen JE, Bantum EO, Criswell K, Bazzo J, Gorlick A, Stanton AL (2014) Representativeness of two sampling procedures for an internet intervention targeting cancer-related distress: a comparison of convenience and registry samples. J Behav Med 37(4):630–641. 10.1007/s10865-013-9509-6 23645145 10.1007/s10865-013-9509-6 PMC3842405

[82] Cheng VWS, Davenport T, Johnson D, Vella K, Hickie IB (2019) Gamification in apps and technologies for improving mental health and well-being: systematic review. JMIR Ment Health 6(6):e13717. 10.2196/13717 31244479 10.2196/13717 PMC6617915

[83] Vajawat B, Varshney P, Banerjee D (2021) Digital gaming interventions in psychiatry: evidence, applications and challenges. Psychiatry Res 295:113585. 10.1016/j.psychres.2020.113585 33303223 10.1016/j.psychres.2020.113585

[84] Berardi C, Antonini M, Jordan Z, Wechtler H, Paolucci F, Hinwood M (2024) Barriers and facilitators to the implementation of digital technologies in mental health systems: a qualitative systematic review to inform a policy framework. BMC Health Serv Res 24(1):243. 10.1186/s12913-023-10536-1 38408938 10.1186/s12913-023-10536-1 PMC10898174

[85] Fleming TM et al (2016) Maximizing the impact of e-therapy and serious gaming: time for a paradigm shift. Front Psychiatry 7:65. 10.3389/fpsyt.2016.00065 27148094 10.3389/fpsyt.2016.00065 PMC4834305

[86] Hampson M ‘Predicting the lifespan of an app’, IEEE Spectrum, 24 Aug 2020

[87] Boucher EM et al (2021) Artificially intelligent chatbots in digital mental health interventions: a review. Expert Rev Med Devices 18(sup1):37–49. 10.1080/17434440.2021.2013200 34872429 10.1080/17434440.2021.2013200

[88] Milne-Ives M et al (2020) The effectiveness of artificial intelligence conversational agents in health care: systematic review. J Med Internet Res 22(10):e20346. 10.2196/20346 33090118 10.2196/20346 PMC7644372

[89] Smith KA et al (2023) Digital mental health: challenges and next steps. BMJ Ment Health 26(1):e300670. 10.1136/bmjment-2023-300670 37197797 10.1136/bmjment-2023-300670 PMC10231442

[90] Khangura S, Polisena J, Clifford TJ, Farrah K, Kamel C (2014) Rapid review: an emerging approach to evidence synthesis in health technology assessment. Int J Technol Assess Health Care 30(1):20–27. 10.1017/S0266462313000664 24451157 10.1017/S0266462313000664

[91] Moher D, Stewart L, Shekelle P (2015) All in the family: systematic reviews, rapid reviews, scoping reviews, realist reviews, and more. Syst Rev 4(1):183. 10.1186/s13643-015-0163-7 26693720 10.1186/s13643-015-0163-7 PMC4688988

[92] Grant MJ, Booth A (2009) A typology of reviews: an analysis of 14 review types and associated methodologies. Health Inform Libr J 26(2):91–108. 10.1111/j.1471-1842.2009.00848.x 10.1111/j.1471-1842.2009.00848.x 19490148

[93] Stoll CRT, Izadi S, Fowler S, Green P, Suls J, Colditz GA (2019) The value of a second reviewer for study selection in systematic reviews. Res Synth Methods 10(4):539–545. 10.1002/jrsm.1369 31272125 10.1002/jrsm.1369 PMC6989049

[94] Michie S, Hastings J, Johnston M, Hankonen N, Wright AJ, West R (2023) Developing and using ontologies in behavioural science: addressing issues raised. Wellcome Open Res 7:222. 10.12688/wellcomeopenres.18211.2 38779420 10.12688/wellcomeopenres.18211.2 PMC11109559

[95] Proudfoot J et al (2011) Establishing guidelines for executing and reporting Internet intervention research. Cogn Behav Ther 40(2):82–97. 10.1080/16506073.2011.573807 25155812 10.1080/16506073.2011.573807

[96] Wykes T, Lipshitz J, Schueller SM (2019) Towards the design of ethical standards related to digital mental health and all its applications. Curr Treat Options Psychiatry 6(3):232–242. 10.1007/s40501-019-00180-0

