# Supplementary material for: Cost-effectiveness of digital health interventions for supporting mental health of children and young people: a rapid review and narrative synthesis
Source: Eur Child Adolesc Psychiatry. 2026 Mar 4;35(6):1699–716. doi: 10.1007/s00787-025-02932-1 (PMC13337612; doi:10.1007/s00787-025-02932-1)
Supplement: Supplementary file 1 — (DOCX 29.0 KB) [file 787_2025_2932_MOESM1_ESM.docx]

**Supplementary Materials**

**Supplementary Material 1: search strategies and results.**

**Search results**

|  | 24/10/2023 search results | 06/05/2025 search results |
| --- | --- | --- |
| Ovid MEDLINE | 740 | 1033 |
| Ovid PsycINFO | 280 | 427 |
| Total | 1020 | 1460 |
| Total after deduplication | 902 | 362 |

**Search strategies**

**Medline (Ovid MEDLINE® Epub Ahead of Print, In-Process & Other Non-Indexed Citations, Ovid MEDLINE® Daily and Ovid MEDLINE®) 1946 to present**

<https://ovidsp.ovid.com/ovidweb.cgi?T=JS&NEWS=N&PAGE=main&SHAREDSEARCHID=2FYodgbhb9skEhdC6DIL6tCSFnf8kpDb0FVSVvp5XyoBM5QWRJ9PClf9TdXMvblmq>

1 Economics/ 27544

2 exp "Costs and Cost Analysis"/ 279272

3 Economics, Nursing/ 4013

4 Economics, Medical/ 9305

5 Economics, Pharmaceutical/ 3162

6 exp Economics, Hospital/ 26220

7 Economics, Dental/ 1922

8 exp "Fees and Charges"/ 31690

9 exp Budgets/ 14349

10 (budget* or "return on investment").ti,ab,kf. 43114

11 (economic* or cost or costs or costly or costing or price or prices or pricing or pharmacoeconomic* or pharmaco-economic* or expenditure or expenditures or expense or expenses or financial or finance or finances or financed).ti,kf. 313618

12 (economic* or cost or costs or costly or costing or price or prices or pricing or pharmacoeconomic* or pharmaco-economic* or expenditure or expenditures or expense or expenses or financial or finance or finances or financed).ab. /freq=2 440527

13 (cost* adj2 (effective* or utilit* or benefit* or minimi* or analy* or outcome or outcomes)).ab,kf. 248743

14 (value adj2 (money or monetary)).ti,ab,kf. 3428

15 exp models, economic/ 16882

16 economic model*.ab,kf. 4753

17 markov chains/ 17106

18 markov.ti,ab,kf. 32985

19 monte carlo method/ 34241

20 monte carlo.ti,ab,kf. 66900

21 exp Decision Theory/ 14348

22 (decision* adj2 (tree* or analy* or model*)).ti,ab,kf. 50089

23 or/1-22 1014899

24 Mobile Applications/ 14983

25 exp Internet/ 106662

26 exp Cell Phone/ 25969

27 exp Computers, Handheld/ 15726

28 Medical Informatics Applications/ 2554

29 Therapy, Computer-Assisted/ 7053

30 (app or apps).ti,ab. 53323

31 (online or web or internet or digital*).ti. 164971

32 ((online or web or internet or digital*) adj3 (based or application* or intervention* or program* or therap*)).ab. 96403

33 (phone* or telephone* or smartphone* or cellphone* or smartwatch*).ti. 31199

34 ((phone* or telephone* or smartphone* or cellphone* or smartwatch*) adj3 (based or application* or intervention* or program* or therap*)).ab. 20532

35 (mobile health or mhealth or m-health or ehealth or e-health or emental or e-mental).ti. 10481

36 ((mobile health or mhealth or m-health or ehealth or e-health or emental or e-mental) adj3 (based or application* or intervention* or program* or therap*)).ab. 7539

37 (mobile* adj3 (based or application* or intervention* or device* or technolog*)).ti,ab. 27059

38 ("social media" or facebook or twitter or instagram or weibo or reddit or snapchat or ("social networking" adj2 (platform* or site* or website*)) or tiktok or wechat or whatsapp or youtube).ti,ab. 52103

39 (texting or "text messag*").ti,ab. 8333

40 "video gam*".ti,ab. 5433

41 24 or 25 or 26 or 27 or 28 or 29 or 30 or 31 or 32 or 33 or 34 or 35 or 36 or 37 or 38 or 39 or 40 435365

42 adolescent/ or young adult/ or child/ 3834236

43 (minor* or boy* or girl* or child* or schoolchild* or pupil* or adolescen* or juvenil* or youth* or teen* or pubescen* or prepub* or pre-pub* or preadolescen* or pre-adolescen*).ti,ab. 2644832

44 ("young adult*" or "young people" or "young person" or "young man" or "young men" or "young woman" or "young women" or student*).ti,ab. 629148

45 Mental Health/ 73169

46 adolescent psychiatry/ or child psychiatry/ 7302

47 Community Mental Health Services/ 19391

48 exp Community Psychiatry/ 2102

49 exp Mental Disorders/ 1550724

50 exp Emotional Adjustment/ 2902

51 exp Mental Health Services/ 110999

52 Affective Symptoms/ 14340

53 exp Intellectual Disability/ 110190

54 Mentally Ill Persons/ 6478

55 Psychopathology/ 9044

56 exp Self-Injurious Behavior/ 90454

57 exp Psychotherapy/ 231947

58 ("mental difficult*" or "mental health challenge*" or "mental health difficult*" or "mental health problem*" or "mental challenge*" or well$being or "mental illness" or "mental health illness" or "mood disorder*").ti,ab. 122404

59 ("mental health" or "attention deficit disorder" or ADHD or "behavio$r disorder*" or "communication disorder*" or "conduct disorder*" or "emotional adjustment*" or "emotional* disturb*" or "intellectual development disorder*" or "learning disorder*" or "psychiatric patient*" or psychopatholog* or suicid* or "thought disturbance*" or psychotherap* or "child psychopatholog*").ti,ab. 496658

60 (psychiatr* or anxiety or depression or suicid* or stress or psychos* or psychotic* or bipolar* or schizophren* or schizoaff* or "eating disorder*" or bulemi* or anorexi*).ti,ab. 2235600

61 42 or 43 or 44 5370157

62 45 or 46 or 47 or 48 or 49 or 50 or 51 or 52 or 53 or 54 or 55 or 56 or 57 or 58 or 59 or 60 3545794

63 23 and 41 and 61 and 62 1611

64 63 1611

65 limit 64 to (english language and yr="2018 -Current") 1033

**PsycINFO 1806 to present**

<https://ovidsp.ovid.com/ovidweb.cgi?T=JS&NEWS=N&PAGE=main&SHAREDSEARCHID=5q2GXokfQOM4Hcvp7ICfmZnmrbqw7hRyUY2BqYESQSR2sHcXXDFDDC274TJsrQVlj>

1 exp "costs and cost analysis"/ 53099

2 economics/ or health care economics/ 31180

3 (budget* or "return on investment").ti,ab. 11694

4 (economic* or cost or costs or costly or costing or price or prices or pricing or pharmacoeconomic* or pharmaco-economic* or expenditure or expenditures or expense or expenses or financial or finance or finances or financed).ti. 51174

5 (economic* or cost or costs or costly or costing or price or prices or pricing or pharmacoeconomic* or pharmaco-economic* or expenditure or expenditures or expense or expenses or financial or finance or finances or financed).ab. /freq=2 116636

6 (cost* adj2 (effective* or utilit* or benefit* or minimi* or analy* or outcome or outcomes)).ab. 30809

7 (value adj2 (money or monetary)).ti,ab. 1221

8 economic model*.ab. 1184

9 markov chains/ 2037

10 markov.ti,ab. 4809

11 monte carlo.ti,ab. 5581

12 exp decision theory/ 2098

13 (decision* adj2 (tree* or analy* or model*)).ti,ab. 11452

14 1 or 2 or 3 or 4 or 5 or 6 or 7 or 8 or 9 or 10 or 11 or 12 or 13 207973

15 mobile applications/ 3517

16 exp internet/ 35059

17 exp mobile devices/ 13119

18 exp computer assisted therapy/ 18119

19 (app or apps).ti,ab. 14082

20 (online or web or internet or digital*).ti. 70765

21 ((online or web or internet or digital*) adj3 (based or application* or intervention* or program* or therap*)).ab. 39027

22 (phone* or telephone* or smartphone* or cellphone* or smartwatch*).ti. 12906

23 ((phone* or telephone* or smartphone* or cellphone* or smartwatch*) adj3 (based or application* or intervention* or program* or therap*)).ab. 6987

24 (mobile health or mhealth or m-health or ehealth or e-health or emental or e-mental).ti. 2347

25 ((mobile health or mhealth or m-health or ehealth or e-health or emental or e-mental) adj3 (based or application* or intervention* or program* or therap*)).ab. 1968

26 (mobile* adj3 (based or application* or intervention* or device* or technolog*)).ti,ab. 10357

27 ("social media" or facebook or twitter or instagram or weibo or reddit or snapchat or ("social networking" adj2 (platform* or site* or website*)) or tiktok or wechat or whatsapp or youtube).ti,ab. 37412

28 (texting or "text messag*").ti,ab. 4263

29 "video gam*".ti,ab. 7227

30 15 or 16 or 17 or 18 or 19 or 20 or 21 or 22 or 23 or 24 or 25 or 26 or 27 or 28 or 29 186864

31 adolescent health/ or late adolescence/ or youth mental health/ 9181

32 (minor* or boy* or girl* or child* or schoolchild* or pupil* or adolescen* or juvenil* or youth* or teen* or pubescen* or prepub* or pre-pub* or preadolescen* or pre-adolescen*).ti,ab. 1201426

33 ("young adult*" or "young people" or "young person" or "young man" or "young men" or "young woman" or "young women" or student*).ti,ab. 727989

34 31 or 32 or 33 1725394

35 exp mental health/ 109197

36 adolescent psychiatry/ or child psychiatry/ 11124

37 community psychiatry/ or exp community mental health/ or exp community mental health services/ 12322

38 exp mental disorders/ 1112617

39 exp emotional adjustment/ 23680

40 exp mental health services/ 85305

41 exp psychopathology/ 46093

42 exp self-destructive behavior/ 60950

43 exp psychotherapy/ 233949

44 ("mental difficult*" or "mental health challenge*" or "mental health difficult*" or "mental health problem*" or "mental challenge*" or well$being or "mental illness" or "mental health illness" or "mood disorder*").ti,ab. 115258

45 ("mental health" or "attention deficit disorder" or ADHD or "behavio$r disorder*" or "communication disorder*" or "conduct disorder*" or "emotional adjustment*" or "emotional* disturb*" or "intellectual development disorder*" or "learning disorder*" or "psychiatric patient*" or psychopatholog* or suicid* or "thought disturbance*" or psychotherap* or "child psychopatholog*").ti,ab. 559584

46 (psychiatr* or anxiety or depression or suicid* or stress or psychos* or psychotic* or bipolar* or schizophren* or schizoaff* or "eating disorder*" or bulemi* or anorexi*).ti,ab. 1146251

47 35 or 36 or 37 or 38 or 39 or 40 or 41 or 42 or 43 or 44 or 45 or 46 1982345

48 14 and 30 and 34 and 47 731

49 48 731

50 limit 49 to (english language and yr="2018 -Current") 427

**Supplementary material 2. Risk of bias assessment**

|  | Andrén et al. 2022 | Andrén et al. 2024 | Aspvall et al. 2021 | Deluca et al. 2021 | Jolstedt et al. 2018 | Kling et al. 2023 | Le et al. 2019 | Lee et al. 2021 | Nordh et al. 2021 | Vargas‐Martínez et al. 2023 | Wasil et al. 2021 | Wright et al. 2020 | Cresswell et al. 2024 | Morrish et al. 2024 | Natsky et al. 2025 |
| --- | --- | --- | --- | --- | --- | --- | --- | --- | --- | --- | --- | --- | --- | --- | --- |
| Is the study population clearly described? | yes | yes | yes | yes | yes | yes | yes | yes | yes | yes | yes | yes | yes | yes | yes |
| Are competing alternatives clearly described? | yes | yes | yes | yes | yes | yes | yes | yes | yes | yes | yes | yes | yes | yes | yes |
| Is a well-defined research question posed in answerable form? | yes | yes | yes | yes | yes | yes | yes | yes | yes | yes | yes | yes | yes | yes | yes |
| Is the economic study design appropriate to the stated objective? | yes | yes | yes | yes | yes | yes | yes | yes | yes | yes | yes | yes | yes | yes | yes |
| Are the structural assumptions and the validation methods of the model properly reported? | N/A | N/A | N/A | N/A | N/A | N/A | N/A | yes | N/A | N/A | N/A | N/A | N/A | N/A | yes |
| Is the chosen time horizon appropriate in order to include relevant costs and consequences? | no | no | no | no | no | no | no | yes | no | no | no | no | no | no | yes |
| Is the actual perspective chosen appropriate? | yes | yes | yes | yes | no | no | no | no | yes | yes | yes | no | no | no | no |
| Are all important and relevant costs for each alternative identified? | yes | yes | yes | yes | yes | yes | unclear | yes | no | no | no | unclear | yes | yes | yes |
| Are all costs measured appropriately in physical units? | yes | yes | yes | yes | yes | yes | yes | yes | yes | yes | yes | yes | yes | yes | yes |
| Are costs valued appropriately? | yes | yes | yes | yes | yes | yes | yes | yes | yes | yes | no | no | yes | yes | yes |
| Are all important and relevant outcomes for each alternative identified? | yes | yes | yes | yes | yes | yes | yes | yes | yes | yes | yes | yes | yes | yes | yes |
| Are all outcomes measured appropriately? | yes | yes | yes | yes | yes | yes | yes | yes | yes | yes | yes | yes | yes | yes | yes |
| Are outcomes valued appropriately? | yes | yes | yes | yes | yes | yes | yes | yes | yes | yes | yes | yes | yes | yes | yes |
| Is an appropriate incremental analysis of costs and outcomes of alternatives performed? | yes | yes | yes | yes | yes | yes | yes | yes | yes | yes | N/A | yes | yes | yes | yes |
| Are all future costs and outcomes discounted appropriately? | N/A | yes | N/A | no | N/A | N/A | N/A | yes | N/A | N/A | N/A | no | N/A | N/A | yes |
| Are all important variables, whose values are uncertain, appropriately subjected to sensitivity analysis? | unclear | unclear | unclear | unclear | no | no | no | yes | no | no | no | no | yes | yes | no |
| Do the conclusions follow from the data reported? | yes | yes | yes | yes | yes | yes | yes | yes | yes | yes | yes | yes | yes | yes | yes |
| Does the study discuss the generalizability of the results to other settings and patient/client groups? | no | no | yes | no | yes | no | no | no | yes | yes | no | no | no | yes | yes |
| Does the article/report indicate that there is no potential conflict of interest of study researcher(s) and funder(s)? | no | no | no | no | yes | no | yes | yes | no | yes | yes | yes | no | yes | no |
| Are ethical and distributional issues discussed appropriately? | no | no | no | no | no | no | no | no | no | no | no | yes | yes | no | yes |
| **Quality** | 72% | 74% | 78% | 68% | 78% | 67% | 67% | 85% | 72% | 78% | 65% | 63% | 78% | 83% | 85% |
